# Autophagic and Apoptotic Pathways as Targets for Chemotherapy in Glioblastoma

**DOI:** 10.3390/ijms19123773

**Published:** 2018-11-27

**Authors:** Cristina Trejo-Solís, Norma Serrano-Garcia, Ángel Escamilla-Ramírez, Rosa A. Castillo-Rodríguez, Dolores Jimenez-Farfan, Guadalupe Palencia, Minerva Calvillo, Mayra A. Alvarez-Lemus, Athenea Flores-Nájera, Arturo Cruz-Salgado, Julio Sotelo

**Affiliations:** 1 Departamento de Neuroinmunología, Laboratorio de Neurobiología Molecular y Celular, Laboratorio Experimental de Enfermedades Neurodegenerativas del Instituto Nacional de Neurología y Neurocirugía “Manuel Velasco Suárez”, Ciudad de México C.P. 14269, Mexico; nasmysr@gmail.com.mx (N.S.-G.); eramed18@gmail.com (Á.E.-R.); phg6219@gmail.com (G.P.); edith.calvillo@gmail.com (M.C.); tlacaelel333@gmail.com (A.C.-S.); jsotelo@unam.mx (J.S.); 2 Hospital Regional de Alta Especialidad de Oaxaca, Secretaria de Salud, Oaxaca C.P. 71256, Mexico; 3 CONACYT-Instituto Nacional de Pediatría, Secretaría de Salud, Ciudad de Mexico 04530, Mexico; racastilloro@conacyt.mx; 4 Laboratorio de Inmunología, División de Estudios de Posgrado e Investigación, Facultad de Odontología, Universidad Nacional Autónoma de México, Ciudad de México C.P. 04510, Mexico; farfanmd@unam.mx; 5 División Académica de Ingeniería y Arquitectura, Universidad Juárez Autónoma de Tabasco, Tabasco C.P. 86040, Mexico; mayra.alvarez@ujat.mx; 6 Departamento de Cirugía Experimental, Instituto Nacional de Ciencias Médicas y Nutrición Salvador Zubirán, Secretaria de Salud, Ciudad de México 14000, Mexico; atheneafloresnajera@gmail.com

**Keywords:** glioblastoma, apoptosis, autophagia, signaling pathways, therapeutic targets, chemotherapy

## Abstract

Glioblastoma multiforme is the most malignant and aggressive type of brain tumor, with a mean life expectancy of less than 15 months. This is due in part to the high resistance to apoptosis and moderate resistant to autophagic cell death in glioblastoma cells, and to the poor therapeutic response to conventional therapies. Autophagic cell death represents an alternative mechanism to overcome the resistance of glioblastoma to pro-apoptosis-related therapies. Nevertheless, apoptosis induction plays a major conceptual role in several experimental studies to develop novel therapies against brain tumors. In this review, we outline the different components of the apoptotic and autophagic pathways and explore the mechanisms of resistance to these cell death pathways in glioblastoma cells. Finally, we discuss drugs with clinical and preclinical use that interfere with the mechanisms of survival, proliferation, angiogenesis, migration, invasion, and cell death of malignant cells, favoring the induction of apoptosis and autophagy, or the inhibition of the latter leading to cell death, as well as their therapeutic potential in glioma, and examine new perspectives in this promising research field.

## 1. Introduction

**Figure 1:**
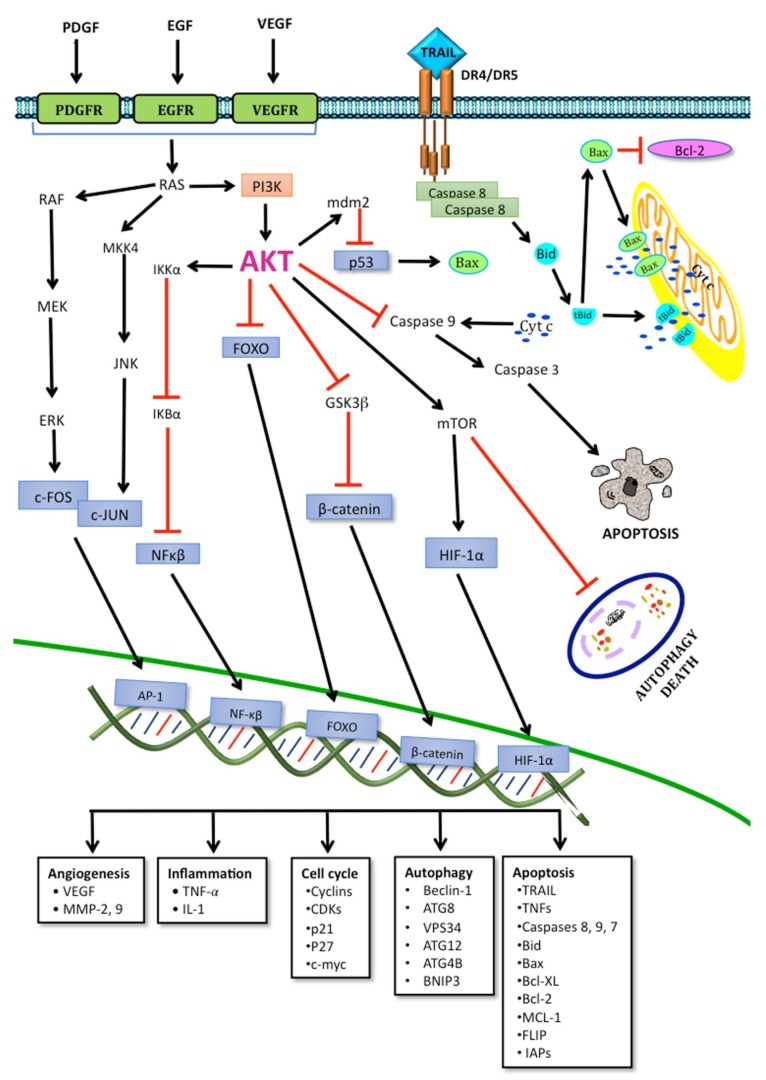
PDGFR-, EGFR-, and VEGFR-mediated signal transduction pathways that are overactivated in glioblastoma. PDGFR (platelet-derived growth factor receptor), EGFR (epidermal growth factor receptor), and VEGFR (vascular endothelial growth factor receptor) are activated at the cell surface by its ligands during tumorigenesis. Activation of these receptors induces several downstream signaling pathways. Among these pathways are the RAS-RAF-MAPK (including ERK, JNK, and p38) and PI3K-AKT-mTOR. These pathways transduce signals into the nucleus to activate transcription factors such as AP-1, NF-κB, FOXO, HIF-1α, and β-Catenin, which regulate the expression of genes that are important for proliferation, cell cycle progression, apoptosis, autophagy, inflammation, angiogenesis, and invasion. The activation of AKT inhibits autophagy death by activation of mTOR, and apoptosis by inactivating p53 and caspase-9. Black arrows (↓) mean activation, red (⊥) mean inhibition.

Brain tumors are a group of highly aggressive and lethal neoplastic diseases [1]. In recent years they reached an approximate incidence of 6–7 cases per 100,000 persons-year [2]. Gliomas are the main neoplastic diseases affecting the central nervous system (CNS), accounting for approximately 50% of all brain neoplasms [3]. Gliomas are derived from glial cells, and the World Health Organization classifies them in several subtypes according to their histological characteristics. Oligodendrogliomas are derived from oligodendrocytes; oligoastrocytomas have a mixed origin, from astrocytomas and oligodendrocytomas; and astrocytomas are derived from astrocytes, being the most frequently observed gliomas. In turn, astrocytomas can be classified according to their degree of malignancy and the severity of their clinical signs into four grades [4]. Grade I astrocytomas exhibit the least malignancy; they are characterized by a low invasiveness, which facilitates their complete surgical removal. Grade II astrocytomas are characterized by a slow progression; however, their surgical cure is hindered by a more extensive infiltration. Grade III astrocytomas show a more rapid progression; they are highly infiltrative and histologically exhibit a marked proliferative activity. Finally, grade IV astrocytomas, commonly called glioblastoma multiforme (GBM), are characterized by poorly differentiated neoplastic astrocytes with cellular polymorphism, nuclear atypia, high mitotic activity, necrosis, vascular proliferation, and thrombosis [5]. These tumors exhibit a great local invasiveness, involving preferential anatomic pathways through the brain parenchyma, subarachnoid space, perivascular space, and white matter tracts; this suggests a variable permissiveness of stroma to the tumorigenic process. This glial tumor infiltration at 1–2 cm from the original tumor mass prevents a total tumor resection after surgery and results in a high rate of tumor recurrence [5]. Gliomas remain refractory to surgical treatment, radiotherapy, systemic or local chemotherapy, and immunotherapy. Epidemiological studies have reported that patient survival is approximately 12–15 months after diagnosis, despite the treatment [6]. The resistance of GBM to a range of therapies is mainly due to a highly mutated genome, which also leads to significant tumor heterogeneity. This involves the overactivation of the RAS/RAF, mitogen-activated protein kinase (MEK), extracellular-signal-regulated kinase (ERK), and phosphoinositide 3-kinase (PI3K)/protein kinase B (AKT)/mammalian target of rapamycin (mTOR) signaling pathways, by activating tyrosine kinase receptors such as the epidermal growth factor receptor (EGFR), the platelet-derived growth factor receptor (PDGR), and the vascular endothelial growth factor receptor (VEGFR), which have been found upregulated in GBM [7,8,9]. These signaling pathways are known to regulate cell proliferation, angiogenesis, migration, and invasion, as well as apoptosis and autophagy (Figure 1); thus, a dysregulation of these signaling pathways favors tumor formation, progression, aggressiveness, and resistance to various therapies [10,11]. One of the major contributors to the observed resistance of GBM to chemotherapy and radiotherapy is the dysregulation of death cell pathways such as autophagy and apoptosis [12], leading to an overexpression of anti-apoptotic proteins (B cell lymphoma 2 (Bcl-2), B cell lymphoma-extra-large (Bcl-xL), and myeloid cell leukemia 1 (Mcl-1)) and cell survival proteins (RAS, PI3K, ERK, c-Jun N-terminal kinase (JNK), AKT, mTOR, and hypoxia inducible factor (HIF-1α)), as well as decreased levels of several pro-apoptotic proteins (Bax, Bad, Bok, NOXA, and apoptotic protease activating factor-1 (Apaf-1)) [13]. There is some evidence that an inhibition of the autophagic process improves the efficacy of irradiation and chemotherapeutic agents, increasing the cytotoxicity of some treatments [14,15]. On the other hand, an increase in autophagy has been linked to a higher therapeutic efficacy in several treatments based on apoptosis induction [16,17,18].

The apoptotic and autophagic processes in glioblastoma are described in this review, along with therapeutic strategies that downregulate signaling transduction mechanisms involved in cell proliferation, survival, invasion, and angiogenesis, to induce apoptosis and autophagic cell death.

### 1.1. Apoptosis

Apoptosis is a type of programmed cell death in which cells are destroyed without releasing noxious substances into the surrounding area. Apoptosis plays a crucial role in corporal development and maintenance by eliminating old, unnecessary, and diseased cells. The human body may replace approximately one million cells per second. If the apoptosis rate is increased, however, several diseases could occur [19]. Characteristically, apoptosis preserves the integrity of the cell membrane until the final phases of death, when the membrane shrinks as cell volume decreases. Since lysosomal contents remain intact, cell fragmentation continues and apoptotic bodies are formed; these are small vesicles linked to the membrane and phagocytized by neighboring cells [20]. These morphological and biochemical processes are primarily mediated by death effectors such as proteases, leading to nuclear and cellular fragmentation. Before these death effector pathways are activated, the life/death balance is modulated by a complex interaction of several death activators. If balance is tipped toward cell death, death effectors start acting [21].

#### 1.1.1. Apoptosis Pathways

**Figure 2:**
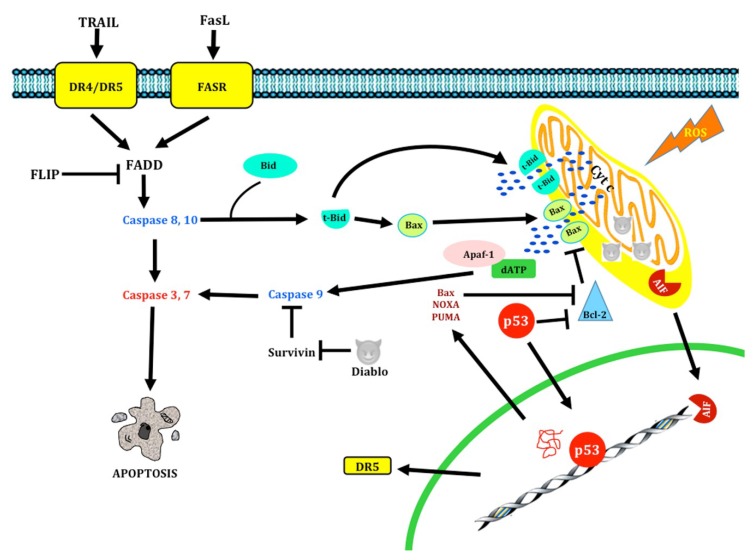
Apoptosis pathways include those initiated by death-receptor ligand or mitochondrial stress (i.e., ROS production). The extrinsic pathway is initiated by the interaction of ligands with death receptors such as TRAIL and Fas. Once activated, they recruit the adaptor protein FADD, which leads to the autoactivation of caspases-8 and -10, which in turn promote the catalytic activation of the effector caspase-3. Another target of caspase-8 is the pro-apoptotic protein Bid, which is hydrolyzed to tBid, inducing Bax oligomerization and mitochondria depolarization with release of cyt c. Along with the activation of caspase-9, these events amplify the apoptotic pathway. The intrinsic pathway involves the permeabilization of the mitochondrial external membrane, which facilitates the cytosolic release of pro-apoptotic proteins such as SMAC/Diablo and cyt c, which are otherwise confined within the intermembrane space. cyt c binds to the Apaf-1 protein, which in turn binds to and activates caspase-9, which is responsible for the activation of the executioners of apoptosis: caspases-3, -6, and -7. On the other hand, SMAC/Diablo inhibits the survivin protein, which binds to and neutralizes caspase-9. Another protein involved in apoptosis regulation is p53, which transcriptionally activates pro-apoptotic genes like Bax, NOXA, and PUMA, directly inhibiting Bcl-2 and favoring apoptosis. Continue arrows (↓) mean activation, truncated arrows (⊥) mean inhibition.

While the molecular mechanisms of apoptosis (the event cascade from the cell surface to the ultimate nuclear changes) still require clarification, several proteins are known to have a key role in regulating programmed cell death [22]. Two main pathways leading to apoptosis have been described: the extrinsic or death receptor pathway, and the intrinsic or mitochondrial pathway [23,24,25] (Figure 2).

#### 1.1.2. Extrinsic pathway

The extrinsic pathway of programmed cell death requires membrane receptors to be activated [24] (Figure 2). These cell death receptors belong to the genic superfamily of Tumor Necrosis Factor receptors (TNFR); they are characterized by a cysteine-rich extracellular domain and a homolog cytoplasmic sequence termed death domain (DD). The Fas receptor (also known as cytotoxicity-dependent protein, CD95, or Apo-1) is a cell surface protein of the tumor necrosis factor/nerve growth factor receptor superfamily [26,27]. Its wide expression in various tissues [28] and in nerve cells [29,30] suggests that the Fas receptor plays a crucial role in apoptosis [31].

FasR is a type-I membrane protein. Its extracellular domain shows two N-glycosylation sites and a cysteine-rich region where the Fas ligand (Fas-L) is bound, as well as an intracellular death domain close to the carboxyl-terminal end of the molecule [26,28,32]. Fas-L belongs to the TNF genic family. It is a type-II, homo-trimeric surface glycoprotein with an approximate molecular weight of 40 kDa; its N-terminal end is embedded in the cytosol and the C-terminal end is extracellularly oriented. When FasR is activated by its cell-associated ligand, the protein forms microaggregates with the FasR death domains. This requires the recruitment of a cytoplasmic adaptor molecule which also features the Fas-associated death domain (FADD). The FADD protein has a single serine phosphorylation site (Ser-194 in humans and Ser-191 in mice) which is essential for cell cycle regulation and for survival and proliferation in some cells [33]. The FADD amino-terminal domain is named death effector domain (DED), and it is responsible for recruiting procaspase-8 [34]. This complex is called death-inducing signaling complex (DISC); the formation of DISC determines procaspase autoactivation by dimerization, yielding the mature form of the protein and the ensuing onset of the apoptotic cascade [35,36]. Then, procaspase-8 is autolytically cleaved to give caspase-8 (the active form), which in turn cleaves and activates cascade effector caspases (caspase-3), fragmenting several substrates until cell death occurs [31]. Caspase-10 can form complexes with FADD and caspase-8 (FADDosome) as well [36]. Recently, it was described that the upregulation of caspase-10 is ATR-dependent and leads to the recruitment of TRAF2. Then, RIP1 ubiquitinates cFLIP, and finally it activates the apoptosis mechanism mediated by caspase-8 [37]. Once activated, caspase-8 could follow two pathways: either activate the caspase cascade as described above, or act on a protein of the Bcl-2 family termed Bid. In its truncated form, Bid can be translocated into the mitochondria and activate the mitochondrial pathway by a yet unknown mechanism. The intrinsic and extrinsic pathways converge at this point [38]. Bcl-2 proteins are clustered into three families: the anti-apoptotic protein family (Bcl-2, Bcl-xL, Bcl-w, Bcl-B, Mcl-1, and Bok A1); the “multidomain”-type pro-apoptotic protein family (Bax, Bak), and the “BH3”-type pro-apoptotic protein family (Bid, Bim, Bad, Bmf, Bik, Hrk, NOXA and PUMA).

#### 1.1.3. Intrinsic Pathway

A second mechanism, which does not depend on death receptors, is the mitochondrial apoptotic pathway (intrinsic pathway) [39,40,41] (Figure 2). Proteins of the Bcl-2 family are involved in the control of apoptosis through this pathway in several cell types, acting either as cell death activators or inhibitors [42]. Bcl-2 is a 26-kDa oncoprotein, primarily located on the inner mitochondrial membrane. A neuroprotector role has been attributed to Bcl-2 against apoptotic cell death [43] by preventing the release of cytochrome c (cyt c) into the cytosol by the pro-apoptotic proteins Bax and Bak, which may affect the permeability of the outer mitochondrial membrane [38]. Once cyt c is released from mitochondria, the apoptosome is formed by assembling the apoptotic protease activating factor-1 (Apaf-1) with procaspase-9. The recruitment of procaspase-9 by Apaf-1 through what is known as the caspase recruitment domain (CARD) requires ATP. Then, procaspase-9 is autolytically cleaved to yield the active caspase-9; the latter detaches from the complex to activate other caspases (caspase-3, -7, and -6), which cut off the contact with neighboring cells, reorganize the cytoskeleton, activate endonucleases, and activate specific proteins that prepare the cell to terminate all metabolic functions. Cyt c release from mitochondria into the cytosol is a key step in regulating caspase activation, since it does not only initiate caspase activation by activating Apaf-1, but it also disrupts the electron transport chain, thus decreasing energy production and increasing the synthesis of reactive oxygen species (ROS) [44]. The apoptosis-inducing factor (AIF) also plays a role in this pathway. It is released from the mitochondrial intermembrane space into the cytosol and then it moves into the nucleus. Once there, AIF binds DNA and triggers DNA destruction and cell death. AIF induces chromatin condensation and the fragmentation of DNA into 50-kDa segments but does not induce oligonucleosomal fragmentation; this stage of DNA damage is called “stage I” condensation [36,45]. Along with cyt c and AIF, other apoptotic factors are released from mitochondria, such as the second mitochondria-derived activator of caspases/direct IAP-binding protein with low PI (Smac/DIABLO), which blocks the action of proteins of the inhibitor of apoptosis (IAP) family. IAP was originally identified as a cell death-inhibiting viral protein; it is characterized by one or more highly conserved 70-amino acid domains. There are five members in this family: cIAP1, cIAP2, XIAP, NAIP, and survivin; they act by blocking apoptosome formation [38]. IAP, which is inhibited by HtrA2/Omi, has also been reported to promote apoptosis [46]. Another compound that is released into the cytosol is the endonuclease G (Endo G). This enzyme is translocated into the nucleus and produces oligonucleosomal DNA fragments. This stage of chromatin condensation is called “stage II” condensation [36]. The p53 gene was first described in 1979, initially identified as a tumor suppressor gene [47]. While the actions of p53 have been widely studied, researchers have focused on its apoptosis-inducing effects. It can induce apoptosis through both the intrinsic and extrinsic pathways. In the extrinsic pathway, death receptors to the tumor necrosis factor-related apoptosis-inducing ligand (TRAIL), including DR5 and DR4, are activated; they are induced in response to DNA damage [47,48]. These receptors bind the p53-induced death-domain-containing protein (PIDD), which activates caspase-8 [49]. In the intrinsic pathway, cytoplasmic p53 can provoke the entry of Bax into mitochondria with the ensuing cyt c release, caspase activation, and apoptosis completion [49]. p53 is a nuclear transcription factor. p53 binds specific DNA sequences and activates the transcription of pro-apoptotic proteins of the family Bcl-2 such as Bax, PUMA, and NOXA (located in the cytoplasm). An increase in PUMA levels can displace p53 from Bcl-xL, to which it is bound, and then activate Bax [49,50,51]. p53 is also able to trigger apoptosis through the cell survival pathway. It has been reported to regulate the phosphatase and tensin homolog on chromosome ten (PTEN), a negative regulator of the PI3K pathway. p53 transactivates the PTEN promoter, thus increasing PTEN expression, which in turn inhibits AKT activation; as a result of AKT inhibition, mdm2 is displaced from p53 and promotes a negative regulatory effect on p53. Additionally, it promotes apoptosis by activating caspase-9 [48,50,51,52,53]. On the other hand, AKT can inhibit apoptosis by phosphorylating pro-apoptotic proteins like Bax, Bad, HtrA2/Omi, and caspase-9 [54]. AKT inhibits the FOXO3 transcription factor, which upregulates the genic expression of pro-apoptotic proteins like Bim and NOXA [54]. AKT also regulates NF-κB through the activation of the IKK complex, favoring the transcription of cell survival genes like Bcl-xL, Bcl-2, c-IAP1/2, and XIAP [54]. Moreover, it has been show that AKT induces apoptosis by mediating the phosphorylation of anti-apoptotic factors like XIAP and Mcl-1, decreasing their stability [55]. It has been shown that JNK is involved in intrinsic or mitochondrial apoptotic signaling. JNK upregulates the expression of apoptosis-specific genes through the activation of transcription factors such as AP-1, p53 and c-myc [56]. JNK also inhibits at Bcl-2 through direct phosphorylation. JNK stimulates the translocation of Bad and Bax to mitochondria with the subsequent release of cyt c into the cytosol through of the phosphorylation (inactivation) of 14-3-3 [56]. JNK also induces the phosphorylation of Bid, generating a truncated bid which translocates to the mitochondria and induces the release of SMAC/DIABLO and OMI [56].

#### 1.1.4. Apoptosis in Glioma

It has been proposed that therapeutic resistance of glioblastoma is due to an upregulation of anti-apoptotic proteins and a downregulation of pro-apoptotic proteins, leading to genetic instability and the activation of oncogenes that favor cell survival and resistance to radiotherapy, chemotherapy, and immunotherapy [57]. The anti-apoptotic protein Bcl-w, which contributes to apoptosis resistance, is upregulated in all glioblastomas, since it inhibits the activation of caspases involved in apoptosis [58,59]. Wick et al. demonstrated that an overexpression of the anti-apoptotic proteins Bcl-2 or Bcl-xL in GBM cell lines increases migration and tumoral invasion through apoptosis resistance [58,59,60]. In recurring glioblastomas, an overexpression of Bcl-2, Bcl-xL, and Mcl-1, and a downregulation of pro-apoptotic proteins like Bax, Bak, Bok, and NOXA have been observed [61,62,63]. A downregulation of Bax has been associated to an adverse clinical outcome [64]. Additionally, higher phosphorylation (inactivation) levels of the anti-apoptotic proteins pBAD and pBIM in GBM patients are linked to a poorer overall survival (OS) [65]. From this discussion it can be inferred that a higher expression of Bax, Bak, Bim, or Bad is likely to confer tumor cells a survival advantage, whereas an increased expression of Bcl-2, Bcl-xL, or Mcl-1 is likely to be associated with a poorer prognosis. The overexpression of pro-apoptotic proteins of the family Bcl-2 gives cancer cells a survival advantage in response to a wide range of apoptotic stimuli by inhibiting the release of mitochondrial cyt c [33]. On the other hand, lower levels of Apaf-1 and procaspase-9 have been observed in a glioma cell line and in GBM patients [66]. However, the differential expression of procaspase-3 observed in GBM patients and GBM cell lines was found to correlate with sensitivity to temozolomide in cell lines and to longer progression-free survival (PFS) in patients [67].

In a similar fashion to the anti-apoptotic Bcl-2 family, Blahovcova et al. demonstrated a dysregulation of the TNF receptor pathway, with a significant reduction of genes such as the members 1A, -10A, and -10B of the tumor necrosis factor receptor superfamily (TNFRSF1A, TNFRSF10A, and TNFRSF10B), FAS, and FADD, as well as a downregulation of pro-apoptotic genes such as CASPASE-8 and CASPASE-7 in human GBM tissue biopsies; the authors suggested that the dysregulation of these genes alters the formation of DISC, and thus the caspase-8 activity and the release of cyt c into the cytosol, affecting apoptosis activation by death receptors and the mitochondrial pathway, favoring tumor resistance to different therapies [66,67]. Wang et al. demonstrated lower transcript and protein levels of FADD in GBM tissues with respect to normal brain tissues [68].

**Figure 3:**
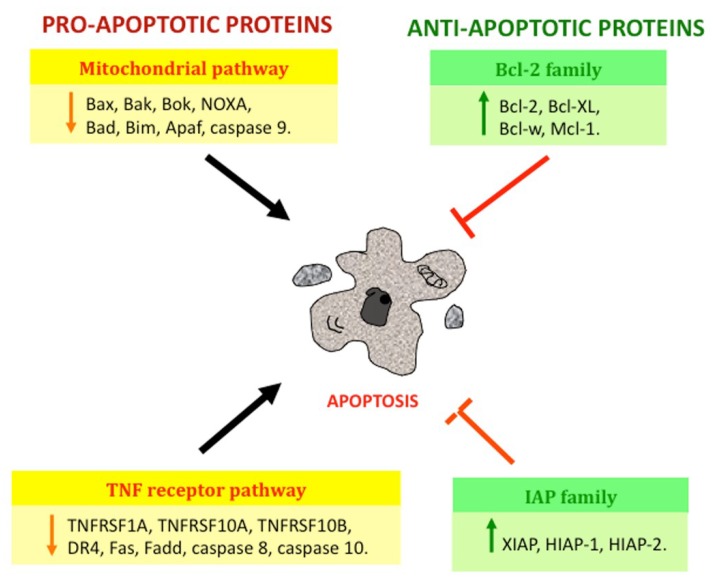
Status of proteins that participate in the apoptotic pathway in glioblastoma. An overexpression of anti-apoptotic proteins such as Bcl-2, Bcl-xL, Bcl-w, Mcl-1, XIAP, HIAP-1, and HIAP-2 has been reported, as well as a downregulation of pro-apoptotic proteins that participate in the mitochondrial apoptotic pathway (Bax, Bak, Bok, NOXA, Bad, Bim, Apaf, and caspase-9) and in the TNF receptor pathway (TNFRSF1A, TNFRSF10A, TNFRSF10B, DR4, Fas, Fadd, and caspase-8 and -9). It has been suggested that the dysregulation of these proteins induces resistance to apoptosis in different therapeutic approaches. Black arrows (↓) mean activation, red truncated arrows (⊥) mean inhibition. Down orange arrows mean downregulation (↓) and up green arrows mean upregulation (↑).

Furthermore, it has been shown that the Fas-FasR pathway is inhibited at the level of caspase-8 activation in glioblastoma patients, and that a lower caspase-8 expression is correlated with a poorer prognosis. Samples from GBM patients that were negative or only weakly stained (<50% of cells) for cleaved caspase-8 had worse cancer-specific overall survival (median was 8.5 months) than patients with tumors that highly expressed cleaved caspase-8 (median was 11.7 months; *p* = 0.0325), irrespective of clinical variables [69]. On the other hand, Ashley et al. reported a low expression of caspase-8 and -10 in U373 glioma cells and glioblastoma tissue [70]. It has been suggested that low levels of caspase-8 and FADD are related to apoptosis resistance via death inducers by TRAIL in glioma [71], since expression levels of the receptors TRAIL-R1 and TRAIL-R2 are increased in biopsy samples from astrocytoma and glioblastoma patients [71]. The expression levels of TRAIL-1 and TRAIL-2 in human glioma biopsy samples were 75% and 95%, respectively [72]. However, Elias et al. reported hypermethylation (epigenetic silencing) of the promoter *DR4* in 60% of diffuse grade II astrocytomas, in 75% of anaplastic astrocytomas, and in 70% of GBM [73]. Additionally, it has been suggested that resistance to TRAIL is due to a higher expression of apoptosis-inhibiting proteins such as IAPs [72]. According to Wagenknecht et al., human malignant glioma cell lines express three members of the IAP family with anti-apoptotic properties: XIAP, HIAP-1, and HIAP-2 [74]. IAPs (particularly survivin) are upregulated in GBM; this upregulation is also associated to a poorer prognosis [11] (Figure 3).

### 1.2. Autophagy

Autophagy is a catabolic process that leads to cellular degradation and the recycling of proteins and organelles by lysosomal digestion. This evolutionarily preserved mechanism is found in mammals, plants, and yeasts [75]. Besides allowing cells to adapt to stressful situations, autophagy regulates cell growth, metabolism, and survival. A basal level of autophagy is considered as cytoprotective, since it contributes to remove misfolded or unnecessary proteins, allowing a balance in cell homeostasis [76]. Moreover, autophagy is essential to mobilize nutritional elements like carbohydrates (glycophagy), lipids (lipophagy), and minerals (ferritinophagy), promoting cell survival by recycling these nutrients [77]. Autophagy is rapidly induced in starvation and several forms of stress, including hypoxia and metabolic, osmotic, and oxidative stress, and even by pathogen infection [78]. In this sense, its dysregulation is involved in processes like tumor suppression, neurodegeneration, ageing, inflammation, and immunity [79,80,81]. Unlike apoptosis, autophagy has been described as a partial chromatin condensation with no DNA fragmenting or blebbing, but with the presence of characteristic autophagic vesicles and increased lysosomal activity [82]. An intercommunication between apoptosis, also known as type-I programmed cell death, and autophagy (type-II programmed cell death) has been proposed: autophagy could increase cell survival by recycling essential elements, but in case of extensive damage it leads to cell death [83,84]. Tumor cells show a decrease in apoptosis, with autophagy increasing cell survival. Under continuous stress, autophagy operates as a cell death mechanism. Thus, autophagy may have either tumor-suppressing or tumorigenic effects. Unexpectedly, it has also been documented that defects in autophagy could be protective and facilitate tumor cell removal [85,86]. Autophagic mechanisms are classified into three types: macroautophagy, microautophagy, and chaperone-mediated autophagy.

Macroautophagy involves the sequestering of substrates by double-membrane vesicles called autophagosomes, which then are fused to lysosomes to allow the degradation of their content. This mechanism is directed by autophagy-related genes (ATG) [87]. First, autophagosomes are formed from the plasmatic membrane, endoplasmic reticulum, Golgi complex, and mitochondria. In the next step, the membrane enlarges and forms phagophores that engulf cytosolic structures and finally forms autophagosomes. After being transported by the cytoskeleton, autophagosomes fuse with lysosomes, forming autolysosomes and allowing the degradation of their content. The resulting elements will be recycled and used to produce energy [88].

In microautophagy, an invagination of the lysosomal or endosomal membrane occurs, with the ensuing degradation of its cargo. Although it is an unspecific process, particular cases like micromitophagy (mitochondria), micropexophagy (peroxisomes), piecemeal microautophagy, and late micronucleophagy (nucleus) have also been described [88,89]. Recently, another classification of microautophagy has been proposed according to the site and morphology of the deformation: protrusion of the lysosome (Type I), invagination of the lysosome (Type II), and invagination of the endosome (Type III) [90].

On the other hand, in chaperone-mediated autophagy, a protein with a KFERQ-like motif is recognized by the heat-shock protein 70-kDa protein (HSC70), which leads to its translocation into the lysosome through the lysosomal-associate membrane protein 2A receptor (LAMP2A) to be degraded [77]. This type of autophagy does not require vesicles [89]. Herein, macroautophagy will be referred to as autophagy in general.

#### 1.2.1. Molecular Mechanisms in Autophagy

The molecular machinery involved in autophagy is quite complex and includes several kinases, phosphatases, and GTPases, which are encoded by autophagy-related genes (ATG); most ATGs have been described in yeasts and their equivalent in mammals have been found [91,92]. At the autophagy onset, proteins or organelles are surrounded by the autophagosome, which is later fused to a lysosome. The fusion of autophagosome and lysosome (autolysosome) allows lysosomal enzymes to hydrolyze the contents of these vacuoles. This process can be subdivided into five steps: initiation, autophagosome nucleation, expansion, completion, docking, and fusion to lysosome. In the initiation, during a homeostatic situation like the presence of amino acids or nutrients, autophagy is inhibited through the phosphorylation of Atg13 by the target of rapamycin complex 1 (TORC1) kinase, preventing the interplay of Atg1 and Atg17. Conversely, in starvation or during rapamycin treatment (an inhibitor of the pathway), TORC1 kinase is inactivated, with consequent hypophosphorylation of Atg13, leading to the interaction of Atg1 with Atg17 and the consequent initiation of autophagy. The Atg1 kinase complex is equivalent to the Unc-51-Like Kinase 1/2 (ULK1/ULK2) complex in mammals, which is associated with the mATG13, FIP200 (equivalent to Atg17 in yeasts), and ATG101 kinases. Similarly, when the ULK1/2 complex is phosphorylated by mTORC1, the pathway is inactivated. When autophagy is induced, mTORC1 is inhibited and dissociated from ULK1/2, which now phosphorylates mATG13 and FIP200 and induces autophagosome formation [93]. On the other hand, ULK1 has been reported to inhibit mTOR [94]. In autophagosome nucleation, cytosolic components are recruited to form the phagophore assembly site (PAS) in yeasts, called omegasomes in mammals. In yeasts, PAS includes a scaffold of Atg17, Atg29, Atg20, Atg24, Atg31, and Atg11, which recruits other proteins like the transport protein particle III (TRAPPIII) and Ypt1 to interact with the coat protein complex II (COPII) and Atg9 vesicles [84,85]. The Atg20/Atg24 complex interacts with Atg1, Atg18, Atg21, and Atg27. Atg20 and Atg24 (both of which are important to form pre-autophagic vacuoles) have PX domains to bind PI3P. The PI3K complex I (which includes Atg6, Atg14, the vacuolar protein sortin 34 (Vps34) and Vps15) leads to the generation the phosphatidylinositol 3-phosphate (PI3P). In mammals, the activation of VPs34, a class-III PI3K, depends on the formation of a complex that includes Beclin-1 (equivalent to Atg6 in yeasts), the beclin-1-associated autophagy-related key regulator or Barkor (Atg14), and Vps15 (p150 in humans) [95]. Beclin-1 is also a key regulator of autophagy: the binding of Beclin-1 to AMBRA-1 (activating molecule in Beclin-1 regulated autophagy-1), the UV irradiation resistance-associated tumor suppressor gene (UVRAG), and Bax-interacting factor-1 (Bif-1) results in autophagy induction. On the contrary, when Beclin-1 binds Bcl-2 or Bcl-xL, autophagy is inhibited. Beclin-1 is also a link with extracellular signals; for instance, the inositol 1,4,5-triphosphate receptor (IP3R) interacts with Beclin-1 after its binding to Bcl-2 [96]. It has also been demonstrated that ULK1 activates the VPs34 complex, which contains ATG14L, by phosphorylating Beclin-1 at Ser-14, favoring the initiation of autophagy [97]. Other modulators have been described, such as Atg18 (WIPI-11 and WIPI-2 in mammals), which binds PI3P so it can be recruited to autophagosomal membranes [98], or Rab5, which recruits Vps34 [99].

**Figure 4:**
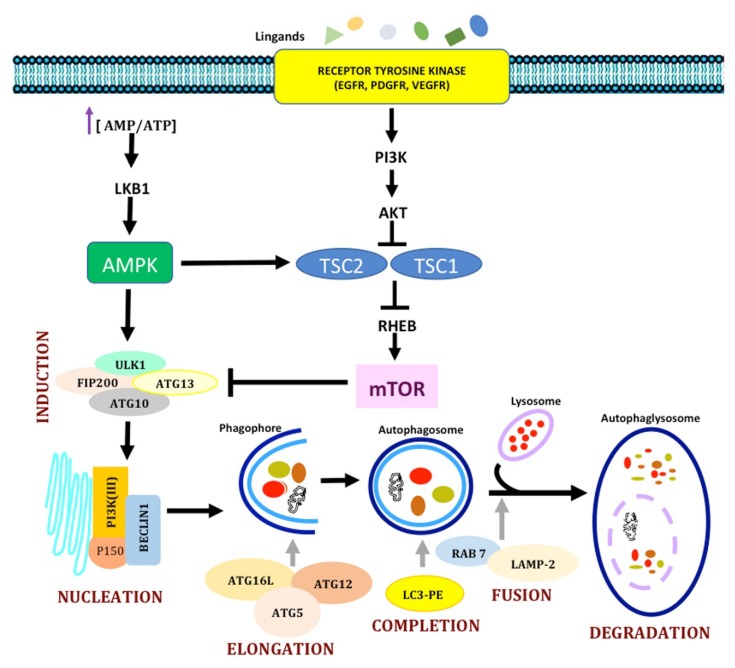
Schematic pathway of autophagy. Autophagy consists of several stages: induction, auto-phagosome nucleation, elongation and completion, fusion to lysosome, and degradation and recycling. Under nutrient-rich conditions, mTOR inhibits autophagy by inactivating the complex kinase-kinase ULK1/2; upon mTOR inactivation due to lack of nutrients, growth factor deprivation, or stress, autophagy is induced; the initial step requires the activity of the PI3K-III/ Beclin-1 complex. The completion and expansion of the autophagosome requires the Atg5/Atg12/Atg16 and LC3-PE proteins. The mature autophagosome fuses with a lysosome to form the autophagosome, which requires the action of several proteins, such as Rab7 and Lamp 1, 2. Black arrows (↓) mean activation and, black truncated arrows (⊥) mean inhibition. Up purple arrow means overexpression (↑) and short gray arrows mean participation in each step.

In the expansion of the phagophore, this initial compartment expands to form the autophagosome. The exact source of the membrane that forms autophagosomes is still unknown. Some hypotheses suggest preexisting intracellular precursor molecules or invaginations of the endoplasmic reticulum, Golgi complex, mitochondria, or even the plasma membrane [82,100]. The Atg9 protein has been proposed to carry membrane components to the phagophore from the trans-Golgi network. The expansion process is regulated by two ubiquitin-like protein conjugation systems: Atg8-phosphatidylethanolamine (PE) and Atg5-Atg12-Atg16. In the first step of the expansion, the ubiquitin-like protein Atg12 is bound to Atg5, and then both proteins form a conjugate with Atg16L1. The Atg12-Atg5-Atg16 complex allows autophagosome expansion and is dissociated when the process is complete. The second step involves the protein microtubule-associated protein 1 light-chain 3 (MAP1-LC3/LC3/Atg8). LC3 is cleaved by Atg4B into the cytosolic isoform LC3-I. Then it is conjugated to phosphatidylethanolamine (PE) by Atg7 and Atg3 to form LC3-II, which remains in the autophagosome until it is degraded in the autolysosomes [101]. Other proteins also facilitate the process; this is the case of Atg10, which interacts with LC3, or the overexpression of Atg3, which facilitates the Atg5–Atg12 interaction. All these complexes lead to the formation of an autophagosome that surrounds the component to be degraded. The autophagosome is transported to lysosomes, where their membranes are fused, forming the autolysosome; the content is then released into the lumen, where hydrolases degrade and recycle cellular components. An important difference between yeast and mammal cells is that in yeasts autophagosomes are formed in PAS, but in mammals they are formed into the cytoplasm and have to be moved toward the microtubule organizing center (MTOC), where lysosomes are located [102]. Some importer proteins like ESCRT, SNAREs, Rab7, and the class-C Vps proteins also participate in the fusion step [103]. UVRAG is also important to regulate autophagosome fusion. UVRAG favors the recruitment of the C-vacuolar protein (C-VPS) to the autophagosome. The interaction of UVRAG with the C-VPS complex promotes a Rab7-GTPase activity, along with proteins like LAMP-1 and LAMP-2; as a result, the autophagosome and the lysosome are fused [104]. Another protein involved is Rubicon, which interacts with Beclin-1, suppressing autophagosome maturation (Figure 4).

Autophagy regulation depends on several signaling pathways, but one of the most studied is the mammalian target of rapamycin (mTOR). This pathway also controls functions like proliferation, cytoskeletal reorganization, and ribosome regulation, depending on the cell energetic status. mTOR regulates protein synthesis by phosphorylating the ribosomal subunit S6 kinase (p70^S6K^), which translates transcripts coding for elongation factors and ribosomal proteins, inducing protein synthesis. mTOR also induces the phosphorylation and inactivation of the inhibitor of the elF4 factor (4E-BP1), activating elF4 to initiate translation [105]. In mammals, mTOR is composed by the complexes TORC1 and TORC2. The mTOR complex I (mTORC1) is formed by the mTOR catalytic subunit, a protein named Raptor (regulatory associated protein of mTOR), GβL (G protein β-subunit-like protein), and PRAS40 (proline-rich AKT substrate of 40 kDa). This complex is also sensitive to rapamycin, an antibiotic produced by *Streptomyces hygroscopicus* that induces autophagy. TORC2 is composed by mTOR, mLST8, rictor, and protor. This rapamycin-insensitive complex regulates cell proliferation and cytoskeleton reorganization. In the case of mTORC1, rapamycin binds the 12-kDa FK506-binding protein (FKBP12), which stabilizes the raptor–mTOR interaction and halts the mTOR phosphorylation activity. In contrast, in the presence of nutrients, amino acids, and growth factors, mTORC1 is activated and inhibits autophagy. It has been reported that mTOR inhibits the initial step of autophagy by phosphorylating (inactivating) Atg13 and ULK1 [94]. On the other hand, mTOR negatively regulates the stability of the protein ULK1 by phosphorylation (inactivation) of AMBRA-1 at Ser-52 [88,97]. Nazio et al. reported that (dephosphorylated) AMBRA-1 induces the polyubiquitination of ULK1 in Lys-63 via an AMBRA-TRAF6 complex (ubiquitin ligase), which promotes ULK1 stability and activity at the onset of the autophagic flow [106]. Nazio et al. also reported that activated ULK1 is a target for the ubiquitin ligase NEDD4L, which induces its degradation via the proteosome; this reduces the amplitude and duration of autophagy [106]. NEDD4L has also been reported to induce the degradation of Beclin-1 [107], and that Cullin3-KHLH20 promotes the degradation of ULK1, Beclin-1, and Vps34 during a prolonged autophagy [108]. In addition, mTOR regulates the transcription of ULK1 by activating elF4E, 4E-BP1, and SK6 [106]. Furthermore, mTOR inhibits the transcription of p62/SQSTM1, VPs11, LC3-II, UVRAG, and WIP, through phosphorylation (inactivation) of the TFEB transcriptional factor at Ser-142 and Ser-211 [109]. It has been shown that phosphorylation at Ser-211 serves as a recognition site for TFEB binding to 14-3-3 and for cytosolic retention [109].

mTORC1 is regulated by the PI3K pathway, activated by the binding of membrane receptors by ligands like growth factors. Activated PI3K converts the plasma membrane lipid phosphatidylinositol-4,5-bisphosphate (PIP2) into PIP3, which recruits the serine/threonine kinases phosphoinositide-dependent kinase 1 (PDK1) and AKT/PKB to the plasma membrane [110]. Phosphorylation of AKT by the rictor-mTOR complex leads to AKT activation and the suppression of autophagy. AKT activation also phosphorylates other proteins such as the tuberous sclerosis complex (TSC), a TSC1/TSC2 heterodimer related to cell growth, considered as the most important regulators upstream of mTOR [88]. Some growth factors lead to ERK activation via RAS/MEK/ERK. ERK and AKT induce the phosphorylation of TSC2, preventing the formation of the TSC2/TSC1 complex and the activation of TORC1. TSC1/TSC2 acts as a GTPase for Rheb, a GTP-binding protein that activates TORC1. AMP-activated kinase (AMPK) and ribosomal S6 kinase 1 (RSK1) are kinases that also regulate mTORC1 by phosphorylating TSC2, thus inducing the activity of the TSC1/2 heterodimer. In conditions of nutrient or energy deprivation, AMPK is activated by LKB1 when the ATP/AMP ratio decreases. LKB1 phosphorylates AMPK at Thr-172 to activate it. When the intracellular concentration of calcium and of cytokines like TRAIL, TNF, or IL-1 increases, the Ca^2+^/calmodulin-dependent kinase kinase (CaMKKβ) and the transforming growth factor-β-activating kinase 1 (TAK1) are activated. CaMKKβ and TAK1 activate AMPK, which induces autophagy by phosphorylating TSC1/TSC2 and inactivating mTOR [111] (Figure 4). On the other hand, it has been shown that AMPK induces the phosphorylation (activation) of ULK1 [94]; it also induces the nuclear translocation of FOXO, which regulates autophagic genes like MAPLC3B, ATG12, ATG4B, VPS3, and Beclin-1 [112]. In Addition, AMPK activates the pro-autophagic Vps34 complex by phosphorylating Beclin-1 at Ser-91 and Ser-94 to induce autophagy [113].

As it can be noted, there is an intricate network of proteins that participate in autophagy; their presence, function, and interactions can be studied by several methods, including transmission electron microscopy (TEM), Western blot, immunoassay, flow cytometry, and fluorescent probes. More precise strategies have been developed to study particular phenomena, and they are expected to provide us with a complete image of autophagy [89].

#### 1.2.2. Autophagy in Glioma

Autophagy has been regarded both as a cell survival and a cell death mechanism in cancer, depending on the cellular context and tumor stage, as well as on the regulation of oncogenes and tumor suppressor genes [84,114]. Autophagy makes available nutrients and energy for cell survival in stressing conditions like hypoxia, lack of nutrients, and therapeutic agents such as irradiation and chemotherapy, allowing tumor progression. On the other hand, autophagy may act as a tumor-suppressing factor by degrading damaged organelles, protein aggregates, and oxidized products, inhibiting the initial phase of the carcinogenic process [86,115].

#### 1.2.3. Autophagy as a Tumor Suppressor

Autophagy has been shown to act as a tumor suppressor in glioma, since the progression of astrocytic tumors is associated with a decrease in autophagic capacity [116]. Furthermore, it has been proved that higher-grade gliomas show lower expression of autophagy-related proteins with respect to lower-grade gliomas [117]: Shukla et al. reported that a lower ULK1/2 expression due to p53 methylation (inactivation) in gliomas favors astrocytic transformation by inhibiting autophagy [118]. Additionally, lower expression or deletion of relevant genes for autophagosome initiation and elongation like FIP200, Beclin-1, UVRAG, Bif1, Atg4c, and Atg5 has been found in GBM [118]. Furthermore, Huang et al. observed lower Beclin-1 and LC3B-II expression levels in GBM, suggesting that a lower induction of autophagy could favor the progression of astrocytic tumors [117]. In addition, high cytoplasmic levels of Beclin-1 have been positively correlated with patient survival and performance (Karnofski classification), while a lower Beclin-1 expression correlated with increased cell proliferation and decreased apoptosis [117]. In vitro studies showed that the overexpression of p72 RNA helicase in T98G and A172 glioma cells and glioblastoma biopsy samples increased the formation of miR-34-5p and miR-5195-3p, which inhibit the expression of Beclin-1, favoring migration, invasion, and apoptosis in neoplastic cells [119].

**Figure 5:**
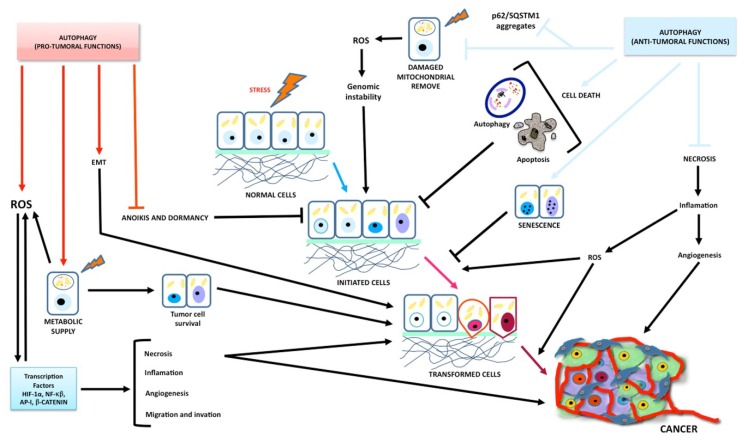
The role of autophagy in the suppressing and promoting tumorigenesis. As a tumor suppressor, autophagy prevents the accumulation of damaged proteins and mitochondria, protecting the cell from oxidative stress and the subsequent genomic instability, necrosis, and inflammatory processes that lead to tumor migration and invasion. If autophagy is excessive, this can lead to type-II programmed cell death, which can induce apoptosis, in addition to activate senescence. In contrast, autophagy sustains tumor survival and growth in adverse conditions (hypoxia and metabolic, osmotic, and oxidative stress) by degrading misfolded or unnecessary proteins and organelles to mobilize nutritional elements like amino acids, lipids, and carbohydrates, promoting cell survival. It also increases oncogenic signals that favor necrosis, inflammation, angiogenesis, migration, and tumor invasion; additionally, it inhibits the anoikis and dormancy. Continue arrows (↓) mean activation and truncated arrows (⊥) mean inhibition.

**Figure 6:**
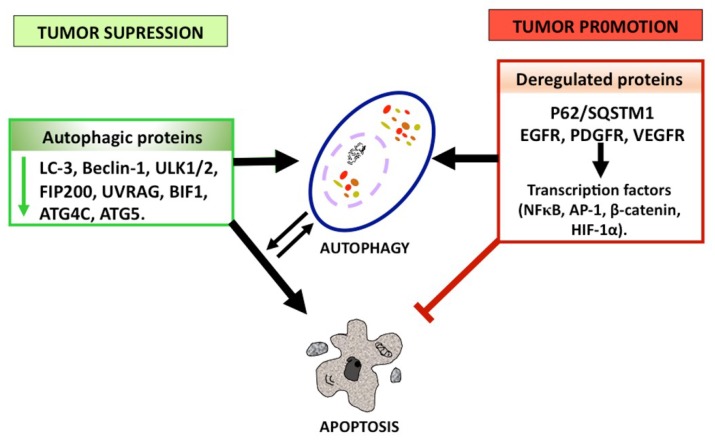
Status of proteins that participate in the autophagy pathway in glioblastoma. A lower expression of autophagy-related proteins like ULK1/2, FIP200, Beclin-1, LC3, UVRAG, Bif-1, Atg4c, and Atg5 has been shown in glioblastoma with respect to lower-grade gliomas, which lead to the progression of astrocytic tumors by inhibiting cell death (both autophagic and apoptotic). Conversely, an increase in autophagy in astrocytic tumors under adverse conditions has been reported to induce tumor survival, progression, and therapeutic resistance through an accumulation of the protein p62, metabolic supply, and activation of transcription factors that regulate the positive expression of genes with an important role in the tumor process. Black arrows (↓) mean activation, red truncated arrow (⊥) means inhibition.

It is noteworthy that higher expression levels of LC3 were associated with an improved survival in GBM patients with poor performance scores, whereas a low LC3 expression correlated with higher survival rates in patients with normal performance scores [120]. Wang et al. reported that a higher miR-33a expression correlated with a poorer prognosis in GBM patients by suppressing the tumor-suppressor protein UVRAG [121]. Additionally, autophagy may suppress tumorigenesis by removing p62-tagged aggregates. The accumulation of p62 in autophagy-defective cells prevents the degradation of protein and damaged organelles, increasing oxidative stress and tumorigenesis [85]. Higher expression levels of p62 have been reported to correlate with tumor grade and a poorer prognosis in adult glioblastoma patients [122]. p62 induces migration and invasion in glioblastoma stem cells by metabolic dysregulation of the RAS/MAPK pathway. In addition, p62 promotes epithelial-to-mesenchymal transition (EMT) by stabilizing TWIST (twist family bHLH transcription factor). In glioma cells, rapamycin reduces invasion and a decrease in the SNAI2 and SNAI1 levels [123]. p62 has also been associated with tumor cell proliferation, survival, and the expression of anti-apoptotic genes like Bcl-2 and Bcl-xL [124]. Autophagy also induces cell death due to inhibited necrosis by limiting the inflammatory response, which favors necrosis-associated tumor growth [125]. Treating the U87 glioma cell line with EGFR-targeted diphtheria toxin (DT-EGF) induced cell death by autophagy, promoting the release of immunomodulatory factors like the high-mobility group box protein 1 (HMGB1), reducing the inflammatory response and inhibiting necrosis [125]. HMGB1 induces an antitumor response by binding the Toll-like receptor 4 (TLR4), activating dendritic cells, killing cancer cells, and inhibiting metastasis [126]. Autophagy induces senescence, further inhibiting tumor growth [127]. Cellular senescence, characterized by a permanently arrested cell cycle, strongly reduces tumorigenesis. Adenovirus strains expressing shMet (an inhibitor of c-met, receptor of the tyrosine kinase of hepatocyte growth factor) reduced the proliferation of U343 glioma cells by promoting autophagy (increasing the levels of Beclin-1, LC3-II, and autophagic vacuoles) and senescence (decreasing SM22 (smooth muscle protein of 22kDa), TGase II (transglutaminase-II), and PAI-1 (plasminogen activator inhibitor-1) mRNA), and blocking the activation of the PI3K/AKT/mTOR signaling pathway; the virus also induced a G_2_/M cell cycle arrest and increased the expression of the cyclin-dependent kinases Cdc2 and Cdc 25C. The authors suggested that autophagy has an antiproliferative effect by inducing a nonapoptotic mechanism [128]. On the other hand, temozolomide has been demonstrated to induce autophagy after senescence in glioma cells, and when autophagy is inhibited by 3MA, senescence is blocked; thus, the authors suggested that autophagy is required for senescence induction [129]. Another mechanism by which autophagy may inhibit tumor progression is apoptosis induction via Atg proteins. The protein atg5 is hydrolyzed by calpain-1 and -2, yielding a truncated atg5; this truncated protein is translocated from the cytosol to mitochondria and associated with Bcl-xL, blocking its anti-apoptotic function and allowing the release of cyt c [130]. Atg5 has also been proposed to interact with the FADD death domain, inducing apoptotic cell death [131]. Thus, atg5 is a signaling regulator linking autophagy and apoptosis; it does not only regulate autophagosome formation by conjugating with atg12, but also it increases the susceptibility to apoptotic stimuli when autophagy is inhibited [131,132]. Jo GH et al. demonstrated that irradiating (10 Gy) glioma cell lines at days 3 and 5 induced autophagy followed by apoptosis, and an atg5 knockdown in U373 and LN229 glioma cells after irradiation significantly diminished both autophagy and apoptosis disregarding caspase activation, indicating that atg5 is required to induce apoptosis [16]. Huang et al. reported that Beclin-1 overexpression in cell gliomas decreased cell viability by inducing apoptosis [133]; the authors propose that Beclin-1 binds and inactivates Bcl-2 and Bcl-xL, allowing the activation of the pro-apoptotic proteins Bax and Bak, which open permeability transition pores, releasing mitochondrial cyt c into the cytosol and subsequently activating caspase-9 and -3, suggesting that Beclin-1 regulates cell death processes like apoptosis and autophagy by binding members of the Bcl-2 family [133] (Figure 5 and Figure 6).

#### 1.2.4. Autophagy as a Tumor Promoter

Irrespective of its role in limiting tumorigenesis, autophagy has been reported not only to activate cell mechanisms favoring cancer cell survival and proliferation, but also to increase resistance to radiotherapy and chemotherapy in apoptosis-resistant tumor cells, thus increasing tumorigenicity [134]. The inhibition of autophagy by irradiation and/or chemotherapeutic agents like cyclophosphamide, tamoxifen, metformin, resveratrol, and N-(4-hydroxiphenyl)-retinamide increases the cytotoxic effect of various treatments [15,135]. Pharmacologic inhibition of autophagy by 3-MA (methyladenine) increases the radiosensitizer effect of WP1066, an inhibitor of the signal transducer and activator of transcription-3 (STAT3) in U251 human glioma cells [136]. The inhibition of autophagy has also been proved to increase the efficacy of the proteasome inhibitor bortezomib, inducing apoptosis by the mitochondrial pathway in U87 glioma cells [137]. Several mechanisms have been proposed by which autophagy favors tumorigenesis: Autophagy favors the survival of tumor cells under hypoxic conditions (insufficient vascularization and limited supply of oxygen and nutrients) in solid tumors like glioblastoma by producing amino acids, fat acids, and metabolic substrates that will allow protein synthesis and energy production for cancer cell survival, proliferation, and resistance [125]. Oxygen concentrations below ~3–0.1% activate the hypoxia-inducible factor 1-alpha (HIF-1α), increasing the expression of BNIP3 (Bcl-2/E1B 19-kDa-interacting protein) and BNIP3L, both of which show an atypical BH3 domain [138]. Bellot et al. suggested that BNIP3/BNIP3L displaces Beclin-1 from the Bcl-2/Beclin-1 or Bcl-xL/Beclin-1 complexes, inducing autophagy [139]. Hu et al. reported increased levels of the BNIP3 protein in U87 and T96G glioma cell lines under hypoxic conditions, inducing autophagy and promoting cell survival, and that the conversion of LC3-I into LC3-II, the degradation of p62, and the expression of BNIP3 were decreased by inhibitors of HIF-1α, promoting cell death by apoptosis [140]. Additionally, the tumor microenvironment may promote the growth of cancer cells via autophagy. Tumor cells lead to an increase in ROS levels, inducing autophagy in fibroblasts; in turn, this promotes glycolysis in stromal cells, increasing the levels of pyruvate, lactate, acetoacetate, and 3-hydroxy-butyrate, which cover the nutritional and energetic requirements of tumor cells, inhibiting apoptosis, and increasing the growth and metastasis of tumor cell [141,142,143]. Oxidative stress induced by cancer cells has been proposed to activate pro-autophagic factors like HIF-1α and NF-κB, inducing an autophagic/lysosomal degradation of caveolin-1 (Cav-1). The tumor suppressor Cav-1 binds and inhibits nitrogen reactive species (NOS), decreasing the decoupling of the respiratory chain and the production of ROS [144]. Decreased Cav-1 levels correlate with higher levels of the monocarboxylate transporter-4 (MCT4) in the stroma and of the lactate transporter (MCT1) in cancer epithelial cells [142]. MCT4 is transcriptionally regulated by HIF-1α under hypoxia or when the ROS levels increase [141,142]. MCT4 exports L-lactate and ketone bodies from glycolytic cells (tumor-associated fibroblasts) [145,146], while MCT1 transporters capture and use them as fuel for tumor growth, invasion, metastasis, and drug resistance. Decreased Cav-1 levels have been reported in glioma endothelial cells under hypoxia [147], while MCT1, MCT4, and the chaperone CD147 are overexpressed in human GBM cells with respect to normal tissues and low-grade astrocytoma cells [148]. Additionally, the expression of MCT1 in the plasmatic membrane has been associated to HIF-1α in hypoxic areas of GBM tumors, and the inhibition of MCT1 by drugs reduced the viability, proliferation, and migration of U251 glioma cells [148]. Autophagy has also been reported to facilitate the dissemination of tumor cells, favoring invasion and metastasis. Autophagy may promote the detachment of tumor cells from the extracellular matrix (ECM), protecting epithelial cells from detachment-induced apoptosis (anoikis) [149]. ECM-detached breast epithelial cells show higher ROS levels and lower ATP levels due to decreased glucose transport rates. To offset detachment-induced stress, the protein endoplasmic reticulum kinase (PERK) is activated in epithelial cells, favoring autophagy, increasing the levels of ATP and prompting an antioxidant response as a support mechanism to promote the survival of ECM-detached tumor cells in ductal carcinoma [150]. Under stress conditions, solid tumors like glioblastoma may escape cell death by inducing autophagy in secondary tumor cells through PERK and the activating transcription factor 4 (ATF4), inducing the expression of autophagic genes like ATG5, ATG7, and ULK, along with hemoxygenase-1, an antioxidant enzyme, to prevent anoikis and favor the survival and migration of tumor cells [151]. AMPK is activated in EMC cell detaching, sustaining growth, and/or preventing apoptosis [152]. Furthermore, under conditions of metabolic stress (lack of growth factors, nutrients, and oxygen) autophagy can improve the survival of cancer cells lacking the pro-apoptotic proteins Bak and Bad or overexpressing the anti-apoptotic proteins Bcl-2 and Bcl-xL, promoting dormancy or quiescence as long as the stress persists and resuming cell proliferation when the stimulus is over [153]. This dormancy period is induced by the loss of integrin β1, which inhibits tumor proliferation but not cell survival, diminishing the focal adhesion kinase (FAK) and the mitogen-activated protein kinase (MAPK), while decreasing the activation of the p38 and elongation factor-2 (elF2α) kinases [154]. By arresting the cell cycle in the phase G_0_ and promoting the stability of the cyclin-dependent kinase inhibitor 1B (p27^KIP1^), LKB1-AMPK induces autophagy and dormancy [155]. According to Magnus et al., dormant cells remain in the brain until alterations in the microenvironment induce tumor progression and recurrence [156]. Glioma cells with low levels of tissue factor show a dormant phenotype in vivo [156]. Brain injury can activate the tissue factor, resulting in chronic coagulation, facilitating the recruitment of inflammatory cells that synthesize cytokines and oxidant products, and allowing dormant cells to express a malignant phenotype [104,156]. Under hypoxic conditions, high levels of the tissue factor in GBM promote genetic alterations that lead to the overexpression of EGFR and EGFRvIII [156,157]. Thus, the inhibition of autophagy could sensitize tumor cells to metabolic stress and prevent resistance to apoptosis. Furthermore, inhibiting autophagy could reduce the availability of nutrients for secondary tumor cells, thus reducing metastasis [158,159] (Figure 5 and Figure 6).

### 1.3. Molecular Correlation between Apoptosis and Autophagy

Depending on the stimulus or the cell type, a sensitization to autophagic death can be induced after apoptosis inhibition, or vice versa; since both processes can induce cell death, there must be a higher-level connection between the apoptosis and autophagy machinery to regulate them. Several studies have demonstrated that apoptosis and autophagy are upstream-regulated by common signaling pathways (RTKs/PI3K/AKT/mTOR and p53). Additionally, proteins taking part in the apoptotic and autophagy machinery (TRAIL, members of the Bcl-2 family, Beclin-1, ATG5, and p62) regulate both processes [26,28,45,48,95,96]. The existence of a link between the molecular mechanisms controlling autophagy and apoptosis was suggested by the finding of an interaction of Beclin-1 through its BH3 domain with Bcl-2 family members (Bcl-2, Bcl-xL, BNIP3, and Bad) [160,161]. The Beclin-1/Bcl-2 complex or Bcl-xL inhibit autophagy in nutrient-rich conditions, since they prevent the interaction of Beclin-1 with the P3IK(III) complex, a kinase responsible for autophagosome formation [85,160]. On the other hand, a lack of nutrients weakens the interaction between Beclin-1 and Bcl-2 or Bcl-xL, and also autophagy induction [162]. It has been described that the Beclin-1/Bcl-2 interaction can be interrupted by BNIP3 and Bad, since both of them displace competitively the protein bound to Beclin-1, promoting autophagy [114]. Another mechanism that prevents Beclin-1 from interacting with Bcl-2 is a phosphorylation of the latter. This process is mediated by JNK, which phosphorylates Bcl-2 at Thr-69, Ser-70, and Ser-87 under conditions of nutrient deprivation [163] or by the Death Associated Protein kinase (DAPK), which phosphorylates Beclin-1 at Thr-119 as part of an ER stress response [164]; in both cases, autophagy is induced. The Bcl-2/Beclin-1 complex is also regulated by pro-apoptotic proteins (Bad, PUMA, NOXA, and Bim), which bind Bcl-2, favoring autophagy [165]. On other hand, Beclin-1 is degraded by caspase-3 to inhibit autophagy [166]. This information suggests that Beclin-1 is regulated by apoptotic components, determining either the inhibition or activation of the autophagic process (cell survival or death) depending on the concentration, activation status, and location of such components. It has been suggested that only ER-bound Bcl-2, and not the mitochondrial type, is able to inhibit autophagy [163]. However, mitochondrial Bcl-2 has been demonstrated to inhibit autophagy by binding and inactivating AMBRA-1 [167]. On the other hand, the permeabilization of the mitochondrial external membrane induced under cellular death conditions has been proved to lead to caspase activation, with the ensuing degradation of pro-autophagic proteins like p62, ATG 4D, ATG5, Beclin-1, and AMBRA-1, producing fragments capable of blocking autophagy and inducing apoptosis by the mitochondrial pathway. Initiator caspases (-8, -9, and -10) and effector caspases (-3 and -6) inhibit autophagy by degrading Atg3, atg5, and Beclin-1 and induce apoptosis by preventing the assembly of the autophagy machinery and producing Beclin-1 fragment [168]. The Beclin-1 fragment has been reported to relocate into the mitochondria and induce the release of pro-apoptotic factors into the cytosol, increasing the apoptotic response [169]. Caspase-3 also hydrolyzes the cysteine-protease atg4D and AMBRA-1, yielding atg4D and truncated AMBRA, which translocate to mitochondria with a BH3-like domain exposed in the carboxyl-terminal end that allows it to bind to Bcl-2 family members and induce apoptosis [170]. Truncated AMBRA-1 prevents autophagosome formation because Beclin-1-PI3K(III) binding was not completed [171]. Caspases-6 and -8 hydrolyze p62, inhibiting autophagy [172].

Some Atg proteins have been suggested to regulate apoptosis. The protein atg5 is hydrolyzed by calpain 1 and 2, rendering a truncated atg5; the latter is translocated from cytosol to mitochondria, associating with Bcl-xL, blocking its anti-apoptotic function, and allowing cyt c release. Additionally, atg5 has been suggested to interact with the FADD death domain, inducing apoptotic cell death [164]. Therefore, atg5 is also a signaling regulator between autophagy and apoptosis, since it does not only regulate the autophagosome formation by conjugating with atg12, but it also increases the susceptibility to apoptotic stimuli when autophagy is inhibited [132]. The atg12-atg3 association has also been reported to regulate cell death by controlling mitochondrial homeostasis [131]. The atg12 induces mitochondrial apoptosis by interacting with and inhibiting Bcl-2 and Mcl-1, leading to Bax activation, the permeabilization of the outer mitochondrial membrane, cyt c release, and activation of effector caspases [166]. Additionally, it has been reported that p62/SQSTM1 induces the activation of caspase-8, leading to apoptosis [173]. On the other hand, UVRAG, a key component of the autophagic machinery, has been reported to induce an anti-apoptotic effect by interacting with Bax and inactivating the mitochondrial translocation of this pro-apoptotic protein, inhibiting the permeabilization of the mitochondrial membrane, cyt c release, and the activation of caspases-9 and -3 [174]. Liu et al. suggested that Beclin-1 inhibits apoptosis by sequestering caspase-8, thus preventing the activation of FasL/FasR [175]. These data demonstrate that several components of the autophagic machinery can regulate apoptosis, independently of their autophagic activity.

**Figure 7:**
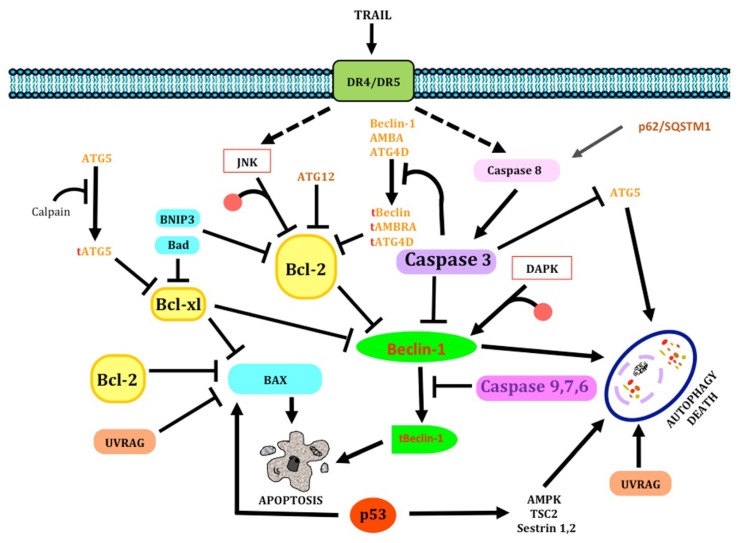
Interplay between autophagy and apoptosis in glioma. Autophagy can induce apoptotic death in several forms: p62/SQSM1 induces the activation of caspase-8, Atg5 is hydrolyzed by calpain, rendering a truncated atg5; the latter is translocated from cytosol to mitochondria, associating with Bcl-xL and blocking its anti-apoptotic function. Atg12 induces mitochondrial apoptosis by interacting with and inhibiting Bcl-2, leading to Bax activation, the permeabilization of the outer mitochondrial membrane, cyt c release, and activation of effector caspases. On the other hand, UVRAG induces an anti-apoptotic effect by interacting with Bax and inactivating the mitochondrial translocation of this pro-apoptotic protein, inhibiting the permeabilization of the mitochondrial membrane, cyt c release, and activation of caspase-9 and -3. These data demonstrate that several components of the autophagic machinery can regulate apoptosis, independently of their autophagic activity. In the other hand, the activation of apoptosis-related proteins such as caspases can inhibit autophagy by cleaving autophagy-related proteins like Beclin-1, p62, Atg4D, Atg3, AMBRA-1, and Atg5, producing fragments capable of blocking autophagy and inducing apoptosis. Another apoptosis and autophagy coregulator is the tumor suppressor p53, which has a dual role: In response to DNA damage, cytosolic p53 induces apoptosis by binding and inhibiting the anti-apoptotic proteins Bcl-2, activating Bax. p53 regulates autophagy by the transcription of AMPK β1 and β2 subunits, ULK2, sestrin 1, and sestrin 2. Black continue arrows (↓) mean activation, black discontinue arrows (↓) mean activation in multiple step, and black truncated arrows (⊥) mean inhibition.

Another regulation point between apoptosis and autophagy is mediated by TRAIL. Thus, defects in apoptosis leading to TRAIL-induced death direct the signaling pathway to a cytoprotective autophagy. On the other hand, inhibiting TRAIL-mediated autophagy initiates a mitochondrial response dependent of caspase-8 that leads to apoptotic death [176]. TRAIL induces apoptosis by binding the death receptors TRAIL-R1/DR4 and TRAIL-R2/DR5, which trigger the homo-trimerization of receptors and the recruiting of cytoplasmic proteins to form the DISC; this complex includes the Fas-associated death domain (FADD), which also binds caspase-8 through its death effector domain, leading to caspase activation. Two different cell death pathways can be deduced after this point. In the first, caspase-8 induces the extrinsic pathway by directly activating the effector caspases-3, -6, and -7. In the second pathway (intrinsic), caspase-8 cleaves the Bid protein, yielding a truncated Bid. The latter is translocated to mitochondria, where it interacts with pro-apoptotic proteins (Bax and Bak); this induces the formation of pores in the outer mitochondrial membrane, causing the release of pro-apoptotic factors (cyt c and Smac/DIABLO). This results in the activation of caspase-9, which activates effector caspases, and in IAP inhibition by Smac/DIABLO, facilitating the process of cell death by apoptosis [40]. TRAIL has also been found to induce autophagy through FADD [177], suggesting that DISC formation from TRAIL could be required for TRAIL to induce autophagy. It has also been suggested that this death ligand induces autophagy by activating AMPK via TAK1, this activates the complex TSC1/TSC2, which in turn inhibits the Rheb protein; the latter activates mTOR, resulting in autophagy inhibition [178]. On the other hand, it has been demonstrated that TRAIL mediates autophagosome formation without the participation of mTOR, by activating JNK independently from caspase-8, which requires FADD and TRAF2 to be activated [179] (Figure 7).

Another apoptosis and autophagy coregulator is the tumor suppressor p53. The activation of this transcriptional factor has a dual role, either negative or positive, on cell death induction [180]. In response to DNA damage, cytosolic p53 is translocated into mitochondria through an apoptotic stimulus and induces apoptosis by binding and inhibiting the anti-apoptotic proteins Bcl-2 and Bcl-xL, releasing Bax and Bak [180]. Additionally, p53 has been reported to induce the transcription of Fas and DR5, leading to the apoptosis extrinsic pathway by activating caspases-8 and -3. p53 also induces the apoptosis intrinsic pathway through the expression of pro-apoptotic proteins (Bax, Bid, PUMA, and NOXA) and Apaf [180], which induce the permeabilization of the external mitochondrial membrane, cyt c release, and apoptosome formation, leading to the activation of caspases-3 and -9 [136]. Another p53-induced factor leading to apoptosis is the lysosomal protein DRAM (DNA damage-regulated autophagy modulator) [181]. It has been suggested that DRAM leads to cell death by permeabilization of the lysosomal membrane, with the subsequent release of cathepsins, which lead to the activation of pro-apoptotic proteins (Bax and Bid), resulting in the permeabilization of the mitochondrial outer membrane and cyt c release [182]. On the other hand, DRAM has been shown to induce apoptosis by promoting mitophagy through DRAM translocation into mitochondria; this process is inhibited by pAKT, which sequestrates DRAM in the cytosol [183]. DRAM has also been described to increase lysosomal acidification, autophagolysosome formation, and autophagic flux [184]. p53 has been reported to regulate autophagy by several mechanisms. Cytoplasmic p53 induces autophagy by AMPK activation, which in turn activates the TSC2/TSC1 complex, leading to mTOR inhibition [185]. It has been demonstrated that p53 forms a complex with LKB1, which activates AMPK [186]. Additionally, p53 regulates the transcription of AMPK β1 and β2 subunits, TSC2, PTEN, and IGF-BP3, which downregulate the IGF/AKT/mTOR pathway [185]. p53 also increases the transcription of ULK2, sestrin 1, and sestrin 2 [187,188]. Sestrin 1 and sestrin 2 bind and activate AMPK [188]. On the other hand, AMPK induces the phosphorylation of p53 at Ser-15 [189]. However, mTOR has been reported to activate PP2A phosphatase, which removes phosphate from the Ser-15 residue in p53, inactivating it [190]. In addition, it has been reported that sestrin 2 binds ULK2 and p62, favoring p62 phosphorylation by ULK2, thus facilitating the autophagic degradation of p62 and its targets [191]. Despite the evidence described above, p53 has been reported to inhibit autophagy. ER stress induces p53 cytoplasmic accumulation, following by its ubiquitination by the E3 ligase HMD2 and the ensuing degradation via proteasome. This favors autophagy through mTOR activation [192]. The deletion of mdm2 or its pharmacologic inhibition increases the stability of p53 and an inhibition of the autophagic process [192]. It has been suggested that autophagy inhibition by cytoplasmic p53 can favor the oncogenic process in some mutants where p53 is mainly located in the cytosol, lacking the capacity of transactivating pro-autophagic genes and being unable to induce apoptosis by the mitochondrial pathway [193] (Figure 7).

### 1.4. Treatment Choices for Glioblastoma

#### Standard of Care

Patients with glioblastoma and anaplastic astrocytoma usually undergo surgical resection of the tumor, radiotherapy (50–60 Gy), and the administration of temozolomide (TMZ). TMZ is a second-generation alkylating agent, suitable for oral administration, with a high penetration capacity into the central nervous system. It is quickly converted into 3-methyl-(triazen-1-yl)-imidazole-4-carboxamide, an active metabolite which methylates some DNA bases (chiefly guanine), causing errors in DNA replication, thus leading to cell arrest in the G_2_/M phase and cell death [194]. A beneficial effect of TMZ in patients with recent GBM diagnosis has been reported; TMZ treatment plus radiation increased median survival from 12.1 to 14.6 months, and survival at 2 years from 10.4% to 26.5% with respect to irradiation alone [194]. TMZ has a tolerable toxicity, with 7% of grade III–IV hematologic toxicity as a concomitant treatment and 14% as an adjuvant. The Food and Drug Administration (FDA) approved TMZ to treat GBM in 2005 [194]. TMZ significantly increases median survival in GBM cases but exhibits a modest therapeutic effect due to the development of chemoresistance by most tumors. The therapeutic efficacy of alkylating agents in cancer is limited by the repairing enzyme methylguanine-O^6^-methyltransferase (MGMT), which repairs DNA by removing the alkyl group from the O^6^ atom in guanine. The therapeutic efficacy of alkylating agents is higher when no MGMT is expressed or when the *MGMT* gene promoter is silenced [195]. Helgi et al. found MGMT hypermethylation in 45% of GBM patients. Additionally, methylation of the *MGMT* gene promoter correlated with a significant increase in survival (21.7 months vs. 15.3 months) and in PFS (10.3 months vs. 5.9 months) in patients receiving TMZ plus radiotherapy [195]. Resistance to TMZ in GBM has neem suggested to be due to an activation of the signaling pathways PI3K/AKT/mTOR and MAPK/ERK, which inhibit apoptosis and activate autophagy. The U-118 glioma cell line, in which the *MGMT* gene is hypermethylated, is more sensitive to TMZ when the PI3K/AKT/mTOR and ERK/MAPK signaling pathways are inhibited [196]. TMZ also induces apoptosis independently of MGMT by activating AKT, which leads to the inactivation of GSK-3β, the stabilization of c-Myc, a decrease in the levels of Bcl-2, and the activation of caspase-3 [197]. The antitumor effects of TMZ involve the induction of autophagy and apoptosis. Würstle et al. reported that TMZ induced autophagy in established (LN18) and primary (pGBM T1 and pGBM T12) glioma cells by inhibiting the EGFR/Beclin-1 complex and Beclin-1 phosphorylation. The authors suggested that TMZ induces the autophagic process by an EGFR-independent mechanism [198]. TMZ induces autophagy (via an increase in LC3) in various glioblastoma cell lines. Treatment with bafilomycin A1 (an inhibitor of vacuolar H^+^-ATPase) to inhibit the fusion of autophagosome and lysosome in the presence of TMZ induces apoptosis by permeabilization of the lysosomal and mitochondrial membranes, with a release of cathepsin B and the activation of caspase-3. Thus, autophagy by TMZ seems to be a self-protection mechanism [158]. Inhibiting the expression of the elongation factor-2 kinase (eEF2 kinase) improved the cytotoxic effect of TMZ in vitro and in vivo by inhibiting autophagy [199]. The eEF2 kinase is involved in protein synthesis and metabolic regulation [199]. TMZ also induces senescence secondary to late autophagy and apoptosis in glioma cells, by inducing guanine methylation and activating the ataxia telangiectasia mutated protein (ATM) [129]. p53 stimulates apoptosis induced by guanine methylation by inducing FasR and activating caspase-8. O^6^MeG also causes apoptosis by the mitochondrial pathway in p53-deficient glioma cells by decreasing the expression of Bcl-2 and activating caspase-9, suggesting that MGMT and p53 could play a role in the sensitivity to TMZ [200]. TMZ also induces apoptosis through the formation of ROS via O^6^MeG, activating LKB2/AMPK; the activation of this pathway stabilizes p53 and inactivates TSC2-dependent mTORC1. p53 activation increases the levels of p21, NOXA and Bax, inactivates the mTORCI signaling, and downregulates Bcl-2; both processes favor apoptosis in primary cultures of glioma cells [201]. Altogether, this information indicates that the cytotoxic effect of TMZ in glioma cells depends on the formation of O^6^MeG and the regulation of several signaling pathways.

### 1.5. Small-Molecule Inhibitors

Tyrosine-kinase inhibitors (TKI) are low-weight molecules capable of binding tyrosine kinase domains in intracellular receptors (EGF, PDGF, and VEGF), blocking competitively the binding of ATP, preventing thus the autophosphorylation of receptors and the activation of intracellular signaling cascades (RAS/RAF/MAPK and PI3K/PTEN/AKT/mTOR). The most widely used TKIs in the clinic to treat GBM are gefitinib and erlotinib (inhibit EGFR), imatinib mesylate (inhibits PDGFR), and sunitinib, vandetanib, and vatalanib (inhibit VEGF).

#### 1.5.1. Erlotinib

Erlotinib has been reported to inhibit EGFR-dependent cellular proliferation in vitro by arresting the cell cycle in the phase G_1_, reducing the levels of cyclin D1, and increasing the expression of the protein p27^kip1^ (inhibitor of cyclin-dependent kinases), which induced the hypophosphorylation of pRB; additionally, it reduces glioma cell migration and invasion by inactivating the ERK, AKT, and RHO pathways [202]. Erlotinib has also demonstrated to inhibit the formation of multicellular spheroids in the U87MG line. Long-term erlotinib treatment downregulates EGFRvIII and invasive markers in glioma cells [202]. The combination of erlotinib and crizotinib (a c-Met pathway inhibitor) diminished importantly tumor volume as well as the phosphorylation of EGFRvIII, Met, AKT, and MAPK in primary human GBM subcutaneous xenografts. Furthermore, the levels of stem cell markers such as Olig 2, Sox2, and Nestin were also significantly diminished by crizotinib [203]. Furthermore, cotreatment with erlotinib and NSC23766 (a RAC1 inhibitor) showed a synergistic effect on human glioma cells; it inhibits cell proliferation, induces cell death by apoptosis and caspase-independent autophagy, and reduces cell survival [204]. A combination of erlotinib and sorafenib (an inhibitor of VEGFR2, PDGF, and RAF) had a synergistic effect on U87MG, LNZ308, and LN428 cells and on Glioma Stem Cells (GSC) by inhibiting the phosphorylation of ERK, AKT, and S6, decreasing the levels of nuclear β-catenin and PMK2, and inducing apoptosis and autophagy [205]. Erlotinib partially improved the survival of nude mice xenografts with GSC [205]. Additionally, erlotinib was proved to induce cell death by autophagy in the U87-MG glioma cell line when administered at higher levels than the therapeutic dose [206]. Despite the antineoplastic effect of erlotinib in preclinical studies, it has shown poor activity in clinical trials. No benefit was observed in patient survival (mean PFS was 1.9 months and survival was 6.9 months) when erlotinib was orally administered as monotherapy [207]. In phase-I/II trials on recurring GBM patients treated with erlotinib, carmustine, or TMZ, the progression-free survival rate after six months (PFS-6) was 11.4% in the erlotinib group and 24% in the carmustine and TMZ groups [208]. In another trial, survival increased from 14.1 months (historic control) to 19.1 months after cotreatment with erlotinib plus TMZ either before or after irradiation [208]. An erlotinib/bevacizumab combination was also tested in patients showing unmethylated MGMT promoter, with discouraging results. No statistically significant difference in survival was observed (mean survival was 13.2 months vs. 12.7 months reported in a historic control) [209]. Another group under study showed a minimal improvement in mean PFS (13.5 months vs. a historic mean of 8.6 months), with no success in mean survival (19.8 months vs. a historic mean of 18 months). Additionally, no relationship was observed between such response and the expression of PTEN, EGFR amplification, or MGMT promoter methylation [210]. When tested [209] in pediatric patients (median age: 10 years, range: 3–19 years), a combination of erlotinib, TMZ, and irradiation failed to improve the disease prognosis; a mean PFS of 10.7 and 6.3 months, and a mean survival of 15.4 and 11.7 months were observed in patients with anaplastic astrocytoma and GBM, respectively [211]. The results of administering erlotinib and temsirolimus to adults suffering from recurrent, high-grade glioma in a phase-II trial were similar to those described above, and no correlation of survival with the presence of the mutation EGFRvIII, EGFR amplification, nor PTEN expression was observed [212]. It has been suggested that the resistance of tumor cells to erlotinib, either as a monotherapy or in combination, is due to the genic expression of the calcium channel, voltage-dependent gamma subunit 4 (CACNG4), fibroblast growth factor receptor 4 (FGFR4), heat shock 70-kD protein 1B (HSPA1B), heat shock 27-kD protein 1 (HSPB1), nuclear factor of activated T cells, cytoplasmic, calcineurin-dependent 1(NFATC1), neurotrophic tyrosine kinase receptor type-1 (NTRK1), RAS-related C3 botulinum toxin substrate 1 (RAC1), smoothened homolog (SMO), transcription factor 7-like 1 (TCF7L1), and transforming growth factor beta 3 (TGFβ3), which regulate positively signaling pathways involved in cancer cell proliferation, migration, invasion, and resistance to apoptosis (JAK-STAT, MAPK, JNK, NF-κB, and WNT) [213].

#### 1.5.2. Gefitinib

Gefitinib has been reported to induce apoptosis in glioma cells by activating the pro-apoptotic protein Bad, with the ensuing mitochondrial translocation of Bax and the activation of caspase-9, which activates caspase-3 [214]. Gefitinib has also been proved to inhibit invasion (dependent of EGFR amplification) and angiogenesis (depending on EGFR status) in glioma tumors implanted in mouse brain slices [215]. On the other hand, Chang et al. showed that at doses lower than 20 µM gefitinib inhibits cell growth and colony formation in U87 and T96G glioma cells, by activating the LKB1/AMPK pathway and inducing autophagy (autophagosome accumulation, LC3-II, and p62 degradation) [216]. A synergic effect of gefitinib with MK-2206 (AKT inhibitor) on anti-glioma activity has been observed in vivo and in vitro by apoptosis induction (survivin downregulation) and autophagy (LC3-II accumulation) at the start of treatment (48 h), and a shift from autophagy to apoptosis (Bim increase) in a late stage of treatment (96 h) by inhibiting phosphorylation of the AKT/mTOR/S6K pathway [217]. A synergic effect on the cytotoxic activity of gefitinib and valproic acid has also been reported in a glioma model by inducing autophagy, with the ensuing generation of ROS and activation of the LKB1/AMPK pathway [218]. Phase I trials have established 500 mg as the maximum tolerated dose (750 mg in patients being concomitantly administered with enzyme inducer drugs, like anticonvulsants). Commonly reported adverse effects include rash, diarrhea, and fatigue [219,220]. In a phase I/II trial, 12.7% of glioblastoma patients showed partial tumor regression after treatment with gefitinib; however, the OS was not better than the historic control [220,221]. Chakravarti et al. reported a PFS-6 of 40% and an OS of 11.5 months in patients with a recent diagnosis of glioblastoma receiving a gefitinib–irradiation combined treatment; no significant differences were found with respect to a radiotherapy-only control (11.0 months) [219]. Helgi et al. found that gefitinib dephosphorylates EGFR with great efficiency, but this is not enough to regulate the signaling pathway, yielding poor results in the mean survival (8.8 months) [222]. Other authors have also demonstrated that administering gefitinib as an adjuvant to radiotherapy failed to show significant changes in patient survival [203,205,221,223]. The resistance of glioma tumor cells to gefitinib has been associated to a loss of expression of PTEN [215] and the phosphorylation (degradation) of the pro-apoptotic protein Bim by ERK, activated via the urokinase plasminogen activator (uPA)/uPAR/MEK [224].

#### 1.5.3. Imatinib

Imatinib inhibits receptors such as PDGFRα and PDGFRβ, and kinase proteins with no receptor role (C-abl and BRCP). Imatinib inhibits cell proliferation, inducing apoptosis in T98G and A172 human glioma cells [225]; it also increases survival rates in mice with intracranial glioblastoma [226] by inhibiting the PDGFR autocrine loop [227]. Imatinib induced cell death by autophagy in U87MG and U373-MG human glioma cells, inhibiting the AKT/mTOR pathway and activating ERK1/2. The induction of autophagy by imatinib is demonstrated by the presence of autophagic vacuoles and an increased expression of LC3 and LC3-II. Autophagy inhibition in its early stages reduces the toxicity of imatinib, while its inhibition in later stages by bafilomycin A1 or RTA 203 increases the cytotoxic effect of the drug, altering the potential of the mitochondrial membrane and causing cell death by apoptosis; thus, an appropriate modulation of the autophagic process could be effective against cancer cells [228]. Cotreatment with chloropyramine increased the toxic effect of imatinib on C6 glioma cells cultured in monolayer or in spheroids, mediated by autophagy followed by apoptosis; in contrast, a chemoresistant effect is observed in imatinib monotherapy due to autophagy induction [229]. A synergic effect was also observed in C6 cells and spheroids treated with imatinib and the cardiovascular drug carvedilol; this combination induced an arrested cell growth induced by autophagy and apoptosis due to mitochondrial damage [230]. The antineoplastic effect of imatinib on glioma cells could be increased by suppressing autophagy, and a finer modulation of this pathway may sensitize tumor cells to chemotherapy. In a phase-I clinical trial, Readon et al. reported that imatinib was well-tolerated when co-administered with TMZ, determining a maximum tolerated dose of 1000 mg/day; a mean PFS and survival time were 41.7 and 56.1 weeks, a discrete benefit based on which the authors propose to conduct phase-II trials [231]. Imatinib has shown a poor antitumor effect on glioblastoma patients in clinical assays, with a PFS-6 lower than 16% and a radiological response below 6% [232]. The low response to imatinib treatment in high-grade glioblastoma may due to a low drug availability in the tumor mass [233,234,235]. In another phase-II trial, tumor biopsies were taken before and after imatinib treatment to test several markers by immunohistochemistry. The radiological response was evaluated by nuclear magnetic resonance on day 7 of treatment, finding a stable tumor in 18 out of 20 patients. Mean survival was 6.2 months (range: 1.1–17.9 months); 13 out of 20 patients received standard treatment, with TMZ and radiotherapy as adjuvants. A decrease in AKT and MAPK phosphorylation, as well as an increase in the expression of the antiproliferative control protein p27, was observed in tumor tissue samples after treatment, demonstrating a biochemical response to it [236]. In a phase II trial where several molecular markers were measured after a combined treatment with imatinib plus hydroxyurea, a positive expression of PDGFRα and a phosphorylated status of the protein were associated to a lower survival. This could be due to the association of the loss of PTEN when PDGFRα is positive [237]. In contrast, the combination of imatinib with nilotinib (second generation TKI) increased the cell migration and invasion in stem cells isolated of patients through the phosphorylation of proteins such as paxillin (PXN), FAK, and p130Cas. The authors suggested that these results could be a significant contributor to the observed lack of clinical efficacy [238].

#### 1.5.4. Sunitinib

Sunitinib acts by inhibiting receptors of the vascular endothelial growth-factor (VEGFR), the colony-stimulating factor-1 (CSF1R), the Fms-like tyrosine kinase-3 (FLT3), the platelet-derived growth-factor (PDGFR), and the stem cell-factor (Kit); these receptors are major angiogenesis inducers. Sunitinib has antiproliferative, antiangiogenic, anti-invasive, and pro-apoptotic effects in vivo by partially inhibiting the activity of FAK and SRC, while increasing the expression of cadherin-11 [239]. Sunitinib decreased the length and density of blood vessels, showing anti-invasive and pro-apoptotic effects due to a partially inhibited phosphorylation of Src at Tyr-418 and FAK at Tyr-397, along with an inhibited activation of caspase-3, in U87MG and GL15 glioma cells and in intracerebral models of GBM [240]. Combined treatments with sunitinib and various drugs capable of potentiating its anti-tumor effects have been tested. An example is the combined administration of sunitinib and chloroquine, which enhanced the cytotoxicity and antiangiogenic capacity of the former, inducing apoptosis by downregulating CD34 and survivin, as well as activating nitric oxide synthase, increasing RNS production and activating caspase-3 [241]. Sunitinib promotes autophagy by increasing the levels of Beclin-1, which was inhibited by chloroquine, with the ensuing increase in p62 [241]. A combination of sunitinib and gefitinib inhibited oncosphere proliferation and growth by blocking phosphorylation in signaling pathways with a role in tumorigenesis like AKT, MAPK, and STAT3, as well as activating apoptosis in vitro [242]. Sunitinib also potentiated the antiproliferative and pro-apoptotic effects of TMZ in vivo; unfortunately, this combination induced vascular resistance by increasing the expression of Ang-1 and Tie 2 [243]. Clinical trials on recurrent primary glioma patients treated with sunitinib failed to show an objective radiological response, with a PFS-6 of 12.5%, median PFS of 2.2 months, and median OS of 9.2 months. Those patients expressing endothelial c-Kit in higher levels have also been proposed to have a higher benefit from this treatment [207]. A modest antitumor response to sunitinib and irinotecan has been observed in glioblastoma patients [244]. No benefits in survival were found when sunitinib was orally administered as a continued monotherapy to patients with a progressive disease who had previously received either the standard treatment or bevacizumab. Additionally, an increased risk of progression and death was observed in those patients who previously received chemotherapy [245]. In another phase II trial, sunitinib was given as a monotherapy to patients who had previously received TMZ; the mean duration of treatment was 1.4 (0.5–13.6) months for the glioblastoma group. The range of stable disease for these patients was 31.3%, PFS6 was 16.7%, mean PFS was 1.4 months, and mean survival was 12.2 months [246]. Sunitinib treatment was given to patients in whom the tumor could not be excised, before and after radiotherapy; sunitinib failed to induce a radiological response neither neurological improvement. Mean PFS was 7.7 weeks, and survival at one year was 0% [219]. The poor response to sunitinib in brain tumors could be explained by a low penetration of the drug into the central nervous system, due to the presence of P-glycoprotein and the breast cancer resistance protein in the blood–brain barrier [247]. Qingyu et al. proposed that the resistance to sunutinib is due to the activation of proliferative signaling pathways mediated by p- c-jun and phospholipase C-γ1 (PLC-γ1), along with the increased expression of ABCG (ATP-binding cassette (ABC) transporters) in U87MG tumors [248].

#### 1.5.5. Vandetanib

Vandetanib is a low-weight compound capable of inhibiting VEGFR, EGFR, and the RET-tyrosine kinase. Vandetanib decreased tumor size in rat intracranial glioma by inducing apoptosis; it also limits angiogenesis by inhibiting VEGF and exhibits a synergistic effect with irradiation both in vitro and in vivo [249]. Vandetanib inhibits signaling pathways activated by EGFRvIII, such as STAT3, AKT, and Bcl-xL, thus reducing cell growth and angiogenesis in glioma cell lines that express EGFRvIII [250]. Cotreatment with Vandetanib and SB203580 (an inhibitor of p38 MAPK) synergistically inhibits cell proliferation in glioma cells [219]. Vandetanib induces autophagy by inhibiting the PI3K/AKT/mTOR pathway, whereas pharmacologic (chloroquine and 3-MA) or genetic (Beclin-1 and ATG7 knockdown) autophagy inhibition boosts the antineoplastic effect of vandetanib through apoptosis by the mitochondrial pathway in glioma cell lines [251]. Chloroquine increases the apoptotic effect of vandetanib on U251 tumor cells in vivo [251]. The combined administration of vandetanib with TMZ or irradiation increased the antineoplastic effect of both therapies [252]. As shown in phase II trials, vandetanib can be safely administrated in combination with irradiation and TMZ, with no undesirable side effects. In this study, nine out of 10 patients exhibited a stable disease in the follow-up. Mean PFS was 8 months, and survival was 11 months [253]. Phase II trials including patients with glioblastoma and anaplastic astrocytoma demonstrated a radiological response to vandetanib. PFS-6 was 6.5% and OS was 6.3 months in the glioblastoma group, while PFS-6 was 7% and OS was 7.6 months in the anaplastic astrocytoma group. One of the most commonly observed adverse effects was rash, found in all patients with a positive response to treatment. Enzyme-inducer antiepileptic drugs did not significantly change the exposition to vandetanib [254]. Lee et al. compared the effect of the TMZ plus irradiation treatment either with or without vandetanib, finding promising results in mean survival (15.9 months in the RT/TMZ group vs. 16.6 months in the vandetanib/RT/TMZ group) and mean PFS (6.2 months in the RT/TMZ group vs. 7.7 in the vandetanib/RT/TMZ group). The ratio of response to treatment was 17.9% in the RT/TMZ group and 25.4% in the vandetanib/RT/TMZ group. A significant increase in the plasmatic placental derived growth factor (P1GF) and a decrease in sVEGFR2 were also observed; both molecules are potential markers to measure the response to anti-VEGF therapy [255]. Pham et al. suggested that the angiogenic response induced by vandetanib enhanced the migration and invasion tumoral through the activation of the TGFβ/TGFβR pathway, with the subsequent stimulation of CXCL12/CXCR4 in VEGFR-positive glioma cells, and that cotreatment with VEGFR, TGFβR, and CXCR4 inhibitors enhanced the therapeutic efficacy in glioblastoma patients [256]. These results suggest that the resistance to antiangiogenic therapy in glioblastoma involves the activation of alternative angiogenic pathways [257].

### 1.6. Targeting Downstream Intracellular Effector Molecules

#### 1.6.1. The RAS/RAF/MAPK Pathway

Farnesyltransferase inhibitors (FTIs) inhibit signaling pathways mediated by RAS and Rho B, like RAS/RAF/MAPK and RAS/PI3K/AKT, since they prevent the post-translational addition of a farnesyl group to the terminal cysteine residue of the CAAX tetrapeptide sequence in several proteins, particularly in p21Ras, expressed by the RAS oncogene. Tipifarnib and lonafarnib are the most widely studied FTIs clinically used to treat glioblastoma.

##### Tipifarnib

Tipifarnib has shown a radiosensitizer effect in SF763 and U87 glioma cells, promoting cell death by necrosis after mitosis by inhibiting the farnesylation of Rho B [258]. Rho B, a member of the GTPase superfamily, is involved in cellular processes like cytoskeletal rearrangement, focal adhesion mechanisms, migration, and invasion. Wang et al. also observed a radiosensitizing effect in the SF763 glioma cell line, mediated by the inhibition of farnesylation of the HJD-2 chaperone protein [259]. It has been shown that HDJ-2 inhibits apoptosis by blocking the translocation of Bax from the cytosol into the mitochondria [258]. Tipifarnib reduces the levels of HIF1α in U87 glioma xenografts by increasing oxygen levels. It also induces the expression and activity of MMP-2, decreasing hypoxia, angiogenesis, and radioresistance. Despite these effects, tipifarnib has shown poor activity in the clinic: a PFS-6 of 12% has been reported in recurrent glioblastoma and a PFS-6 of 9% was found in anaplastic astrocytoma [258]. In phase I trials, the antineoplastic activity of tipifarnib in GBM was unsatisfactory [260]. Tipifarnib administration before irradiation did not improve the survival of GBM patients [261]. In another phase II trial, a combined treatment of tipifarnib plus irradiation exhibited a progression time of 23.1 weeks and an OS of 80.3 weeks in GBM patients. An overexpression of the fibroblast growth factor receptor 1 (FGFR1) was correlated with shorter progression times, while the overexpression of the integrin αvβ3 and of FGFR1 were correlated with a lower OS [262]. In another phase II trial, tipifarnib was administered to recurrent, malign glioma patients, stratified by the concomitant administration, or not, of enzyme-inducer antiepileptic drugs. That study showed a better response in those patients who did not receive anticonvulsants (PFS was higher than 6 months, and PFS-6 was 16.7% vs. 6.4%). However, this may be due to the fact that the group taking enzyme-inducer drugs had received a higher number of chemotherapy rounds than the other group [263].

##### Lonafarnib

Lonafarnib was reported to inhibit cellular proliferation in anaplastic astrocytoma cell lines and in GBM-xenografted mice [264]. Lonafarnib reduces cellular viability and induces an arrested cell cycle in the G_2_ stage by decreasing the phosphorylation of MAPK in the U251/E4 MG cell line (which overexpresses EGFR) [265]. Lonafarnib enhanced the cytotoxic effect of irradiation and TMZ in orthotopic GBM tumors and in neurospheres derived from human GBM cells [266]. The pro-autophagic effect of lonafarnib has been reported in various cell lines. Lonafarnib and other FTIs induce cell death by autophagy in solid tumor cells and inhibit caspase activity [267]. The mechanisms by which FTIs induce autophagy are currently being studied, but alterations in the RAS/PI3K/AKT/Rheb/mTOR pathway could be involved, along with ROS formation and DNA damage. Lonafarnib has proved to reduce the activation of mTOR in a dose-dependent manner [267]. A combined treatment of lonafarnib-TMZ showed a PFS-6 of 38%, and a median PFS of 3.9 months. The median disease-specific survival was 13.7 months in 27% of patients with recurrent glioblastoma [268].

#### 1.6.2. Targeting PI3K/AKT/mTOR

PI3K/AKT/mTOR pathway plays a vital role in survival, proliferation, and progression, migration and invasion of glioblastoma. Drugs known to inhibit mTOR, like temsirolimus, sirolimus, and everolimus, are currently being studied in clinical trials.

##### Temsirolimus

Temsirolimus is an analogue of rapamycin, capable of binding the FK-binding protein-12 (FKBP-12) to inhibit mTOR [269]. Temsirolimus has antiproliferative effects in mouse low-grade gliomas [270]. Co-administered with perifosine (an AKT inhibitor), temsirolimus has shown antiproliferative and anti-apoptotic effects independently of the PTEN status, related to an inhibition in the PI3K/AKT/mTOR pathway [271]. The inhibition of mTOR by temsirolimus increases the cytotoxic and pro-apoptotic activity of PENAO (an inhibitor of adenine nucleotide translocase) on DIPG (diffuse intrinsic pontine gliomas) cells through the generation of reactive oxygen species, ATP depletion, and an increase in the activity of AMPK, with the subsequent inhibition of the PDGFRa /PI3K/mTOR and HSP90 signaling pathways [272]. Chandrika et al. demonstrated that mTOR inhibitors like temsirolimus and torin significantly reduce the expression levels of mesenchymal markers (fibronectin, vimentin, and YKL40) and neural stem cell markers (Sox2, Oct4, nestin, and mushashi1) induced by the tumor promoter phorbol-myristate-acetate in LN-18 glioma cells, through the dephosphorylation of the transcriptional factor STAT3 [273]. In a phase II study, temsirolimus failed to exhibit antineoplastic effects in GBM patients, either as a monotherapy or combined with erlotinib. Additionally, the combined therapy increased the incidence of adverse effects. No positive correlation was found between survival and markers like EGFR amplification, mutation in EGFRvIII, or PTEN status [212]. Patients with recurrent GBM showed no radiological response after a temsirolimus/bevacizumab combined treatment (median PFS was 8 weeks and OS was 15 weeks) [274]. The combined administration of temsirolimus and sorafenib (a RAS, VEGF, and PDGF inhibitor) did not improve survival in recurrent GBM patients either (mean PFS was 8 weeks and progression-free patients at 6 months was 0%) [275]. Another phase II trial compared the effect of combined temsirolimus/radiotherapy with radiotherapy/TMZ treatment in patients who did not show hypermethylation of the MGMT promoter. Mean follow-up was 33 months in the temsirolimus group and 32 months in the TMZ group. Mean survival rates were 14.8 months and 16.0 months, respectively. Tumor samples from the patients were analyzed for molecular markers, finding that those expressing mTOR Ser-2448 phosphorylated were associated with a longer survival (mean survival was 17.8 months for temsirolimus-treated patients showing p-mTOR Ser-2448 vs. 13.1 months in negative cases for the marker) [276].

##### Everolimus

Everolimus potentiates the capacity of TMZ to induce cell death by autophagy in U87 glioma cells [277]. Everolimus decreases the activity of the enzymes lactate dehydrogenase and choline kinase α, reducing the levels of lactate and phosphocholine by transcriptional inhibition of HIFα in glioma models, both in vitro and in vivo [278]. Olmes et al. reported that the combination of everolimus and palbociclib (an inhibitor of CDK4/6) significantly alters the metabolism (it inhibits aerobic glycolysis), with the subsequent induction of apoptosis in glioma-initiating cells in vitro and in vivo; furthermore, everolimus significantly increases the penetration and accumulation of palbociclib in the brain, inactivating the proteins P-glycoprotein and BCRP1 [279]. Everolimus and LBT613 (an inhibitor of RAS) exert a synergistic effect, inhibiting proliferation, migration, and invasion in the glioma cell lines U87MG and U373MG [280]. A combined treatment with everolimus and AEE788 (which inhibits EGFR and VEGRF) was more efficacious to induce apoptosis and cell cycle arrest both in vitro and in vivo than any of them as a monotherapy [281]. A synergistic cytotoxic effect was observed by treating U87MG human glioma cells with everolimus (codenamed RAD001) plus the Delta-24-RGD adenovirus. A combination of the drug and the virus induced autophagy in vitro and in vivo by promoting the expression of Atg5, a key step to transform LC3-I into LC3-III. An increase in the mean survival of mice with glioma (80%) was reported [282].

In a phase I trial, a combined everolimus/radiotherapy treatment with TMZ as an adjuvant was well tolerated by GBM patients [283]. By adding everolimus and bevacizumab to the standard irradiation/TMZ therapy, GBM patients showed a median PFS of 11.3 months and a median OS of 13.9 months [284]. However, a combined gefitinib/everolimus treatment did not show therapeutic efficacy over the historic control, although a radiological response was observed in 33% of recurrent glioblastoma patients [285].

##### Sirolimus

Sirolimus is known to arrest the cell cycle in the G_1_ stage and induce apoptosis in several glioma models [286]. A combined sirolimus/erlotinib treatment significantly reduced cell proliferation and induced apoptosis in GBM cells by blocking the PI3K/AKT/mTOR pathway, independently of PTEN [287]. Sirolimus, formerly known as rapamycin, inhibits mTOR and induces autophagy. Both the induction of cell death by autophagy and the inhibition of cell growth by rapamycin have been observed primary GBM and U87MG cell cultures. Additionally, inhibited growth and decreased tumor size were observed in GBM-transplanted naked mice after rapamycin treatment [286]. Inhibiting mTOR enhances the effect of sirolimus, and those cell lines with mutated PTEN (U87MG, A172, and D54) are more sensitive to this treatment than cells featuring non-mutated PTEN (LN229) [288]. The capacity of sirolimus to inhibit mTOR and induce autophagy was confirmed in U87MG, T98G, and U373 MG glioma cells, where inhibition of the PI3K/AKT/mTOR pathway led to the induction of autophagy without activating apoptosis. Additionally, sirolimus had a synergistic effect with LY294002, an inhibitor of PI3K, and UCN-01, an inhibitor of AKT [289]. Sirolimus induced autophagy in glioma stem/progenitor cells (GSPCs) and promoted differentiation of such cells both in vitro and in vivo [290]. Rapamycin has also showed synergistic effects with the oncolytic adenovirus OBP-405. Rapamycin boosts the autophagy induced by OBP-405 in GBM U87MG and U251-MG cell lines [291]. Additionally, radiosensitivity has been restored in radiotherapy-resistant AMC-HN-9 cells by a combined rapamycin/PP242 treatment (being the latter an inhibitor of mTOR), possibly mediated by autophagy and senescence induced by activation of p53 and RB-E2F [292]. Hsu et al. demonstrated that a combination of TMZ, chloroquine, and sirolimus induces apoptosis in a synergistic way on U87 cells, through the permeabilization of the mitochondrial membrane and the subsequent release of cathepsin B [293]. However, in a phase I trial, a combined sirolimus/gefitinib treatment showed only a partial radiological response in GBM patients [294]. In another study, a combination of sirolimus and erlotinib failed to show a radiological response, with a mean follow-up of 69.3 weeks, a median OS of 33.8 weeks, mean PFS and PFS-6 of 6.9 weeks and 3.1%, respectively, in recurrent glioblastoma patients; interestingly, a positive correlation was found between patients showing hyperlipidemia and a better outcome [295]. A combined vandetanib/sirolimus treatment in GBM patients led to a partial radiological response, with a PFS-6 of 15.8% [295]. The poor response of GBM patients to mTOR inhibitors could be due to the activation of AKT by the IGF-IR/IRS-1/PI3K pathway [296].

### 1.7. Bcl-2 Inhibitors

The apoptotic pathway via mitochondrial cyt c release is regulated chiefly by members of the Bcl-2 family [297]. Anti-apoptotic proteins like Bcl-2, Bcl-xL, and Mcl-1 are often overexpressed in glioblastoma, allowing cellular proliferation and the resistance of tumor cells to various therapies [57]. The pharmacologic inhibition of these proteins has been experimentally used to reduce glioblastoma.

#### 1.7.1. ABT-737

It is a BH3-mimetics. These compounds bind BH3 grooves, selectively and with a high affinity, in proteins of the Bcl-2 family, including Bcl-2 and Bcl-xL, but not Mcl-1, being capable of inducing apoptosis and autophagy [204]. ABT-737 has been tested as a monotherapy in various glioblastoma cell lines, finding a limited sensitivity to treatment [298]. It has been suggested that the pro-apoptotic protein Mcl-1 mediates ABT-737-induced resistance to apoptosis [299]. However, the drug has shown promising effects when combined with other treatments. When combined with bortezomib (a proteasome inhibitor), ABT-737 induced apoptosis and a strong activation of caspase-3 and, to a lesser extent, of caspases-8 and -9 in the LN229 and LN18 cell lines, which have an intact PTEN expression. In the U87 and LNZ308 cell lines, with a PTEN deficiency, the effect was not significant. By exploring the anti-apoptotic pathway AKT, which is common in the PTEN and Bcl-2 pathways, AKT1 was found to be an important mediator to explain the resistance to treatment in cells with PETN deficiency [300]. ABT-737 has proved to have a synergistic effect when co-administered with cucurbitacin-I, significantly inhibiting the proliferation of the cell lines U87, U87-EGFR-WT, U87-EGFRvIII, T98G, LN18, and LNZ308. No active forms of the caspases-3, -7, -8, or -9, nor of PARP, were detected; the administration of zVAD-fmk, a caspase inhibitor, did not alter the effect of the combined treatment, suggesting a mechanism of cell death independent of caspase activation. When observing the nuclear morphology of the cells treated with this drug combination, the presence of fragments or lobulated nuclei was observed in association with micronuclei, a trait corresponding to a mitotic catastrophe. On the other hand, the suppression of Aurora kinase A or Aurora kinase B, or the expression of survivin in the same cell lines increased significantly the sensitivity to ABT-737 treatment [300]. Dinaciclib is a CDK inhibitor whose effect is not related to p53, p14ARF, nor the amplification status of PTEN or EGFR in some glioma cell lines. It also inhibits Mcl-1 expression in glioma cells in a dose-dependent manner, by proteasomal degradation; thus, it sensitizes tumor cells to favor the effect of ATB-737, causing the loss of mitochondrial membrane potential and inducing apoptosis, as demonstrated by the cleavage of caspase-3 and PARP, cyt c, and Smac/DIABLO cytosolic accumulation, as well as Bax activation [301]. The multikinase inhibitor sorafenib is also a Mcl-1 inhibitor, useful in sensitization to ATB-737 treatment, showing a higher apoptosis induction in LN229 and U87 glioma cells. This effect was replicated by adding a STAT3 inhibitor (WP-1066), suggesting that the inhibition of the STAT3/MCL1 axis is a relevant target in the sensitization to apoptosis by these molecules [302]. Another Mcl-1 and survivin inhibitor, YM-155, increases the sensitivity of glial cells to ATB-737 treatment; fragments of caspase-3, caspase-7, and PARP were found, along with Bak and Bax activation and a loss of membrane potential in mitochondria. The sensitivity to treatment was inversely proportional to the EGFR activation status, as observed in the U87-EGFRvIII cell line, which was resistant [303]. Treatment of glioma cells with ATB-737 and TRAIL has proved to be effective to induce apoptosis by cleaving Bid into tBid, activating caspase-8, and facilitating the accumulation of tBid on the mitochondrial membrane after ABT-737 neutralized Bcl-2 and Bcl-xL. This combination was also found effective on U87MG and U138MG cells, which express low Bim and Bak levels [304]. In a study where the U251 cell line was treated with TMZ, a significant increase in the expression of mRNA coding for Bcl-2 was observed in surviving cells, with no changes in Bax nor Bak expression. No improvement in apoptosis induction was observed by treating those cells with ABT-737 [305]. Bcl2L12 is an inhibitor of the activation of postmitochondrial effector caspases that is overexpressed in glioblastoma, playing an anti-apoptotic role; it features a dominion similar to BH3 in its structure, which interacts with Bcl-xL and Bcl2. Mutations in the h1 or h2 residues of this dominion lead to a reactivation of pro-apoptotic markers in the U87MG cell line, either treated with TMZ or untreated. ABT-737 can attenuate that dominion [306].

#### 1.7.2. Gossypol

Gossypol is another BH3-mimetics; it binds the BH3 dominion in Bcl-xL, Bcl-w, Bcl-2, and Mcl-1, inactivating them. It is a polyphenolic, liposoluble compound isolated from cottonseed oil. It has antiproliferative effects against human glioma cell lines, both in vitro and in vivo [307]. It has been demonstrated that the (−)gossypol enantiomer (AT101) significantly increases cell death by mitochondrial dysfunction and autophagy induction in apoptosis-resistant human glioma cell lines; cell death was inhibited by a knockdown of pro-autophagic proteins like Beclin-1 and Atg5, and it was increased by a mTOR knockdown [308]. TMZ and ionizing radiation have been reported to increase AT101-induced cell death by autophagy in U343 (MGMT negative) and U87 (MGMT positive) glioma cells [18,307]. The authors suggest that the inhibition of members of the Bcl-2 family by gossypol induces a respiratory burst, which stimulates autophagy [18]. It has been reported that under mitochondrial stress, pro-apoptotic proteins like Bcl-2 increase the synthesis of glucagon and antioxidant enzymes, preventing ROS production and the ensuing cell death [309]. Gossypol has also been proved to induce autophagy by freeing BECN-1 from its inhibitory interaction with Bcl-2 [307]. On the other hand, the use of inhibitors of the HSF-1/HSP90/BAG3 pathway increases the antineoplastic effect of AT101, reactivating caspase-dependent apoptosis in the U252 and U343 glioma cell lines through a downregulation of Bcl-2 and Mcl-1 and an increase of Bax and mitochondrial dysfunction [310]. It has been proposed that BAG3 is a co-chaperone of HSP70 induced by the HSF-1 transcription factor, and that it critically regulates Bcl-2 and Mcl-1 levels by inhibiting the proteasomal degradation of anti-apoptotic proteins, increasing the resistance of cancer cells to various therapies [311]. Festa et al. have suggested that the complex BAG3-HSP70 sequestrates Bax, inhibiting Bax mitochondrial translocation and apoptosis by the mitochondrial pathway [312]. On the other hand, Jarzabek et al. demonstrated that a TMZ/gossypol combination has antiproliferative, anti-invasive, and anti-apoptotic effects in several glioma tumor models [313]. The authors suggested that these effects are due to the inhibition of the urokinase-type plasminogen activator and metalloproteinase-2 through the inhibition of Bcl-2 by gossypol and of TGFβ2 by TMZ [313]. Additionally, gossypol was evaluated in recurrent, malignant glioma patients, observing a good tolerance to the drug, with a low but measurable antineoplastic response [314].

#### 1.7.3. Berberine

Another Bcl-2 inhibitor, berberine is an alkaloid extracted from the roots of the plants *Berberis aristata* and *Tinospora cordifolia*. It has been used as a broad-spectrum antibiotic and exhibits a wide range of pharmacologic and biologic activities, such as antimicrobial, anthelmintic, anti-inflammatory, and antioxidant [315]. In glioblastoma T98G cells, berberine inhibits the activity of Bcl-2, increasing the levels of Bax, caspase-9, and caspase-3 [316]. As a monotherapy, berberine inhibits the proliferation of human glioblastoma T98G cells. An interesting mechanism of action of this molecule is the inhibition of the cell cycle progression, observed as an increase in the percentage of cells in the G_1_ stage and a decrease in the number of cells in the stages S and G_2_/M. Additionally, a decrease in the expression of cyclin D, cyclin E, CDK2, and CDK6 was observed; these proteins are involved in the regulation of the cell cycle. Berberine treatment increased the expression of Bax and decreased that of Bcl-2, stimulating the release of cyt c and the activation of caspase-3 and PARP [317]. Berberine has been observed to cause DNA damage in C6 cells as a dose-dependent effect; on the other hand, it induces ROS production in an exposition-dependent manner, as well as an increase in Ca^2+^ production [318]. In another study, apoptosis induction mediated by ER stress was observed in berberine-treated T98G cells [316]. A combination of berberine and arsenic trioxide (As_2_O_3_) significantly reduced cellular migration and invasion when incubated with glioma C6 and U-87 cells for 12 and 24 h. Immunolocalization assays showed a structural rearrangement and a morphological change of cytoskeleton actin. The cotreatment inhibited the translocation of PKCα and PKCε from the cytosol to the membrane; both molecules are involved in tumor migration and survival. PKC can transmit signals to the nucleus through MAPK-mediated cascades, activating ERKs that in time induce the production of transcription factors like c-myc and jun. Both c-myc and c-jun levels were significantly reduced after berberine/As_2_O_3_ treatment [319]. On the other hand, it has been demonstrated that berberine induced autophagy in glioma cells through the activation of AMPK, with subsequent inactivation of mTOR and an increase in the levels of phosphorylated ULK1 [320].

### 1.8. TRAIL/TRAILR Pathway Activators

A promising strategy to induce apoptosis in glioma cells by the extrinsic pathway, with a possible amplification of the intrinsic pathway, involves reactivating the signaling pathway TRAIL/TRAILR, which induces the formation of DISC and the ensuing activation of apoptosis initiator and effector caspases, with a low toxicity at the peripheral and cerebral levels. TRAIL is generally found in a homo-trimeric form bound to a plasmatic membrane; it can be processed, yielding a trimeric soluble form (sTRAIL), which retains its activity [321]. TRAIL activates apoptotic death by noncovalently binding the receptors R1/DR4 and TRAIL-R2/DR5 [321].

#### 1.8.1. Recombinant TRAIL, sTRAIL, and Anti-DR4 and -5 Antibodies

Recombinant TRAIL is not active as a monotherapy in many GBM primary cell cultures and xenograft models in vivo. Unfortunately, despite the promising preclinical results, its application in clinical trials has been disappointing, due in part to its short half-life, instability in vivo, and tumor resistance to TRAIL. It has been proposed that resistance to TRAIL, either intrinsic or acquired, could be overcome by co-administering TRAIL-based agents with chemotherapeutics, irradiation, or other novel drugs [322]. Co-administration of TRAIL with bortezomib (a proteasome inhibitor) to TRAIL-resistant GBM cell lines and to primary tumors showed a synergistic effect to induce apoptosis through the inactivation of the NF-κB transcription factor [323]. Doxorubicin and MS-275 (a HDAC inhibitor) were also identified as TRAIL-sensitizing agents for GBM [324,325]. To cope with the poor TRAIL half-life and the resulting low concentration of sTRAIL in the tumor, neural stem cells that express sTRAIL were built. Neural stem cells are known to exhibit an extensive tropism for GBM and migrate toward outgrowing microsatellites. Thus, a potent anti-GBM effect of TRAIL-transduced neural stem cells has been observed [326]. A combination of these neural stem cells with TMZ synergistically inhibited GBM outgrowth [327]. Analogously, the use of human umbilical cord blood-derived mesenchymal stem cells (UCB-MSC) transduced with sTRAIL led to higher survival rates of GBM-bearing mice [327]. Zhang demonstrated that the overexpression of miR-7 and TRAIL in MSC has a synergistic tumor suppression effect in TRAIL-resistant GBM cell lines and in U87-xenotransplanted mice, by inhibiting XIAP [328]. Additionally, TRAIL-overexpressing endothelial progenitor cells could migrate toward glioma cells and activate the extrinsic apoptotic pathway by activating the DR4 and DR5 receptors and caspases-8 and -3 [329]. Human sTRAIL has been demonstrated to induce a potent pro-apoptotic activity when selectively binds a predefined tumor cell surface antigen, not showing any effect on normal cells. The EGFR-targeted TRAIL fusion protein scFv425:sTRAIL simultaneously blocks EGFR-mitogenic signaling, thereby sensitizing tumor cells to apoptosis through the TRAIL-receptor signaling in the A172 glioblastoma cell line [330]. Additionally, the aggressiveness of GBM has been demonstrated to be due to an overexpression of the human multidrug resistance protein 3 (MRP3). Wang et al. constructed a novel fusion protein, named scFM58-sTRAIL, in which the MRP3-specific scFv antibody M58 was fused to the N-terminus end of human sTRAIL. The construct showed a significant apoptosis-inducing activity toward MRP3-positive GBM cells in vitro [331]. Another experimental strategy used to activate the apoptotic TRAIL pathway involves the production of anti-DR5 antibodies. Human mAbs against DR5 (E11, H48, and KMTR2) showed cytotoxicity, as well as tumor regression effects in human glioma cells by inducing apoptosis, with no effect on normal astrocytes, by downregulating c-FLIPL and AKT [332].

#### 1.8.2. Taxol

Taxol is a drug used to treat several types of cancer, including glioblastoma. Taxol acts on the TRAIL death receptor, which in turn activates caspase-8. It can also translocate Bax into the mitochondria and release cyt c, which activates caspase-9 [333]. To minimize its severe side effects, low taxol doses (100 nM) along with Bcl-2 siRNA have been assayed [334]; both Bcl-2 mRNA and the expressed protein were decreased in human glioblastoma U138 and U251 cell lines. This decrease in Bcl-2 increased the number of apoptotic cells with respect to cells treated with taxol and siRNA only. The TRAIL death receptor has been found to induce resistance to cell death by apoptosis. It is crucial to study the molecular mechanisms involved and devise potential therapies capable of inhibiting proteasome, such as taxol or bortezomib combined with TRAIL, to decrease resistance and the tumor size. This was the case of glioblastoma U87 and T98 cells, where incubation with bortezomib (a proteasome inhibitor) along with TRAIL increased cell death with respect to bortezomib and TRAIL alone. This rise in cell death is due to the increase in TRAIL expression, which activates caspase-8; in turn, the latter produces a truncated Bid, which remains in cytosol since bortezomib prevents the proteasome from degrading it; Bid increases Bax and caspase-3 expression. This combination provides a new approach to activate cell death in glioblastoma [335]. TRAIL induces apoptosis even when Bcl-2 is overexpressed in the tumor, since the former activates caspase-8, Bid, and caspase-9 in glioblastoma LN-229 cells when cultured with TRAIL [336]. Other proteins involved in the resistance to antitumor treatment are BH3-type proteins in the Bcl-2 family (BNIP3). In normal astrocytes, BNIP3 are located inside nuclei, where they prevent apoptotic death; this mechanism is used by glioblastoma tumors to induce resistance. The DR5 receptor plays a relevant role, along with BNIP3, in such resistance. BNIP3 decreases DR5 expression in tumors, leading to resistance to TRAIL-mediated death by preventing the activation of caspase-8. This provides a novel therapeutic target to increase the sensitivity to treatment in glioblastoma tumors [337].

#### 1.8.3. TIC10/ONC201

Another approach to restore the sensitivity of tumor cells to the extrinsic pathway is the use of compounds that induce the endogenous production of TRAIL. TIC10/ONC201 (imipiridone) has been reported to have an antineoplastic effect on tumor cells by increasing TRAIL and DR5 transcription through the activation and nuclear translocation of FOXO3a, inactivating the kinases ERK and AKT [338]. Tu et al. demonstrated that TIC10/ONC201 activates apoptosis by increasing the stability of the pro-apoptotic protein Bim, by inhibiting Bim phosphorylation (degradation) at Ser-69 by ERK [339]. TIC10/ONC201 has also proved to induce the activation of the pathway eIF2α/ATF4, which positively regulates the transcription of pro-apoptotic proteins like TRAIL, DR5, and the CCAAT/enhancer binding protein homology protein (CHOP), which can induce apoptosis [340]. On the other hand, TIC10/ONC201 inactivates the JAK/STAT signaling pathway and the transcriptional factor NF-κB, as well as the proinflammatory cytokine IL-32β [4]. It has been proposed that inhibiting the JAK/STAT pathway contributes to inactivate the kinases ERK and AKT [341]. JAK phosphorylates receptors with tyrosine kinase activity, which in turn activate the kinases ERK and AKT via the Ras signaling [342].

TIC10/ONC201 has shown cytotoxic activity independent of p53 in various glioma cell lines [343], including TMZ-resistant lines like T98G [1]. In mice intracranially xenotransplanted with the SF767 glioma line, a single administration of TIC10 doubles the overall survival in mice with respect to controls [338]. A combination of TIC10 and bevacizumab was proved to increase three times the survival of mice with intracranial glioma tumor with respect to controls [338]. The antiproliferative and pro-apoptotic effects of TIC10 on glioma lines are synergized when combined with ABT263 (a BH3-mimetic and Bcl-2 and Bcl-xL inhibitor); such effects are independent on the activity of caspase-8 and DR5, but dependent on Mcl-1 degradation and on downregulation of Bag3 and the deubiquitinase Usp9X, which allows the activation of the mitochondrial apoptosis pathway by the pro-apoptotic proteins BAK and Bax. In vivo (a subcutaneous glioma model in proneural transgenic mice), a combination of TIC10 and ABT263 inhibited glioma cell growth by inducing apoptosis, with no side effects [344]. The authors suggested that apoptosis via death receptors was not observed in vivo because the concentrations of TIC10 used were not high enough to activate TRAIL and DR5 transcription [344]. In a phase-II trial including 17 recurrent glioblastoma patients, the administration of 625 mg of TIC10/ONC201 every 3 weeks yielded an OS of 10.4 months, with an OS9 of 53% and OS6 of 71%; the compound was well tolerated. The authors suggest that TIC10/ONC201 can be used as a monotherapy in recurrent glioblastoma.

## 2. Conclusions

**Figure 8:**
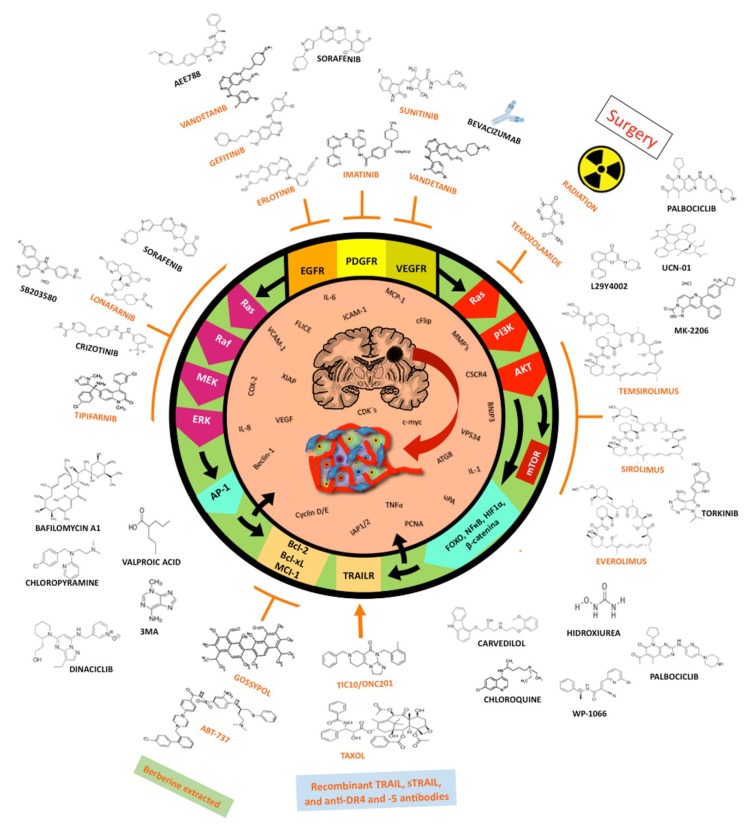
Several therapeutic modalities have been assayed to potentiate cell death in glioblastoma, including activators of apoptotic autophagy cell death as well as inhibitors of the autophagy process. The drugs most widely used in the clinic to treat GBM are temozolamide with radiation and surgery; tyrosine-kinase inhibitors (TKIs) (gefitinib and erlotinib); imatinib mesylate (an inhibitor of PDGFR), sunitinib, vandetanib, and vatalanib (VEGF inhibitors); tipifarnib and lonafarnib (RAS/RAF/MAPK pathway inhibitors); temsirolimus, everolimus, and sirolimus (PI3K/AKT/mTOR pathways inhibitors); Bcl-2 inhibitors (ABT-737, gossypol, and berberine); and TRAIL/TRAILR pathway activators (recombinant TRAIL, sTRAIL, and anti-DR4 and -5 antibodies, taxol, and TIC10/ONC201). All these compounds increase their antineoplastic activity when more than one are administered or when they are combined with other antineoplastic compounds like crizotinib (a c-Met pathway inhibitor), sorafenib (an inhibitor of VEGFR2, PDGF, and RAF), bafilomycin A1, chloroquine and 3MA (autophagy inhibitors), MK-2206 (an AKT inhibitor), valproic acid (an activator of the LKB1/AMPK pathway), SB203580 (an inhibitor of p38 MAPK), palbociclib (an inhibitor of CDK4/6), AEE788 (which inhibits EGFR and VEGRF), bevacizumab (antibody against VEGF), LY294002 (an inhibitor of PI3K), UCN-01 (an inhibitor of AKT), torkinib (an mTOR inhibitor), and dinaciclib (a CDK inhibitor). Continue arrows (↓) mean activation, and truncated arrows (⊥) mean inhibition.

Gliomas are the most difficult tumors to treat due to their high invasive capacity and frequent resistance to apoptosis, which favors tumorigenesis, and their inherent resistance to the standard chemotherapy/radiotherapy approach, being autophagy, which usually is an alternative cell death mechanism, a factor that allows tumor cell to overcome various anticancer agents. In vivo and in vitro studies have demonstrated that apoptosis and autophagy are upstream-regulated by common signaling pathways like RTKs/PI3K/AKT/mTOR and p53. Some inhibitors of RTKs/PI3K/AKT/mTOR increase the sensitivity of apoptosis-resistant glioblastoma cells to pro-apoptotic and/or pro-autophagic drugs (Figure 8). The initial response to the current treatments is often transient, and most tumors eventually progress to higher-grade, highly invasive gliomas. While negative results are due in part to a poor penetration of the blood–brain barrier by several molecules, the presence of CD133-positive cells with an increased expression of the drug-transporter protein BCRP1 (breast cancer resistance protein 1) and MGMT, as well as anti-apoptosis proteins like Bcl-2 and Bcl-xL, and apoptosis inhibitors like FLIP (FLICE (caspase-8) inhibitory protein), cIAP1, and survivin could also play a role in the resistance to treatment in glioblastoma. Determining the molecular mechanisms underlying the capacity of glioma stem cells to survive in a quiescent state may allow us to develop novel therapeutic strategies resulting in more efficacious and less toxic therapies for this devastating type of tumors. The development of combination therapies targeting both the bulk of tumor cells and tumor-propagating glioma stem cells is the most promising approach for future glioma treatments, by manipulating the signaling pathways that control apoptosis and autophagy, given their capacity to regulate the death of neoplastic cells. Essential signaling pathways like the PI3K/AKT cascade and those related with proteins of the Bcl-2 family are involved in the processes regulating both death mechanisms. Apoptosis contributes invariably to the death of cancer cells, while autophagy plays a Janus-like role for neoplastic cells, which adds uncertainty to its manipulation in antitumor treatments. Both constructive and interfering interactions have been reported between apoptosis and cell death by autophagy. Autophagy may promote or regulate cell death by apoptosis, but in certain circumstances the autophagic pathway may start only when apoptosis is inhibited. Thus, it is crucial to elucidate the molecular mechanisms involved in apoptosis and autophagy, as well as the interconnections between both forms of programmed cell death. Although several antitumor agents intended to regulate these signaling pathways have been used in the clinic, the search for more effective anticancer drugs is urgent. In conclusion, the great advances in our understanding of the molecular details of apoptosis and autophagy will provide a better knowledge on fundamental aspects of anticancer therapies. As those molecular mechanisms are clarified, more refined therapies against this disease will be devised, and new avenues will open in the near future to investigate and select more efficient drugs to treat malignant neoplastic diseases like glioblastoma.

## Abbreviations

3 MA	methyladenine
Activating factor-1	Apaf-1
Activating transcription factor 4	ATF4
Activator of transcription-3	STAT3
Adenosine triphosphate	ATP
AMBRA-1	Activating Molecule in Beclin 1-Regulated Autophagy
AMP-activated kinase	AMPK
Apoptosis-inducing factor	AIF
Autophagy-related genes	ATG
Ataxia telangiectasia mutated protein	ATM
ATP-binding cassette (ABC) transporters	ABCG
Bax-interacting factor 1	Bif-1
B cell lymphoma 2	Bcl-2
B cell lymphoma-extra-large	*Bcl*-*xL*
BH3-type proteins in the Bcl-2 family	BNIP3
Binding Protein Homology Protein	CHOP
Ca^2+^/calmodulin-dependent kinase kinase	CaMKKβ
Calcium channel, voltage-dependent gamma subunit 4	CACNG4
Calcineurin-dependent 1	NFATC1
Caspase recruitment domain	CARD
Caveolin-1	Cav-1
Central nervous system	CNS
c-Jun N-terminal kinase	*JNK*
Coat protein complex II	COPII
Colony-stimulating factor-1	CSF1R
C vacuolar protein	C-VPS
Cytochrome c	cyt c
Death effector domain	DED
Death Domain	DD
Death-inducing signaling complex	DISC
Diffuse Intrinsic Pontine Gliomas	DIPG
DNA damage-regulated autophagy modulator	DRAM
Elongation factor-2	elF2α
Elongation factor-2 kinase	eEF2 kinase
Epidermal growth factor receptor	EGFR
EGFR-targeted diphtheria toxin	DT-EGF
Extracellular matrix	ECM
Farnesyltransferase inhibitors	FTIs
Fas-associated death domain	FADD
Fas ligand	Fas-L
Fas receptor	FasR
Fibroblast growth factor receptor 4	FGFR4
FK-binding protein-12	FKBP-12
Fms-like tyrosine kinase-3	FLT3
Focal adhesion kinase	FAK
Food and Drug Administration	FDA
G protein β-subunit-like protein	GβL
Glioblastoma multiforme	GBM
Glioma Stem Cells	GSC
Glioma stem/progenitor cells	GSPCs
Heat shock cognate 71 kDa protein	*Hsc70*
Heat shock 27-kD protein 1	HSPB1
Heat shock 70-kD protein 1B	HSPA1B
High-mobility group box protein 1	HMGB1
Human multidrug resistance protein 3	MRP3
Inhibitor of apoptosis	IAP
Inositol 1,4,5-triphosphate receptor	IP3R
Lysosomal-associate membrane protein 2A receptor	LAMP2A
Methylguanine-O^6^-methyltransferase	MGMT
Mitogen-activated protein kinase	MAPK
Monocarboxylate transporter-4	MCT4
Neurotrophic tyrosine kinase receptor type-1	NTRK1
Nitrogen reactive species	NOS
Overall survival	OS
Paxillin	PXN
Phagophore assembly site	PAS
Phosphatidylinositol 3-phosphate	PI3P
Phosphatidylinositol-4,5-bisphosphate	PIP2
Phosphatidylinositol-3,4,5-trisphosphate	PIP3
Phosphatidylethanolamine	PE
Phospholipase C-γ1	PLC-γ1
Platelet-derived growth factor receptor	PDGR
Proline-rich AKT substrate of 40 kDa	PRAS40
Progression-free survival	PFS
Protein endoplasmic reticulum kinase	PERK
RAS-related C3 botulinum toxin substrate 1	RAC1
Reactive oxygen species	ROS
Ribosomal S6 kinase 1	RSK1
Second Mitochondria-derived Activator of Caspases	*Smac*
Direct IAP-Binding protein with Low PI	*DIABLO*
Serine/threonine kinases phosphoinositide-dependent kinase 1	PDK1
Stem cell-factor	Kit
Smoothened homolog	SMO
Target of rapamycin complex 1	TORC1
Temozolomide	TMZ
Tyrosine-kinase inhibitors	TKI
Transcription factor 7-like 1	TCF7L1
Transforming growth factor beta 3	TGFβ3
Transforming growth factor-β-activating kinase 1	TAK1
Tensin homolog on chromosome ten	PTEN
Toll-like receptor 4	TLR4
Transport protein particle III	TRAPPIII
Tumor Necrosis Factor receptors	TNF
Tumor Necrosis Factor receptors	TNFR
Tumor necrosis factor-related apoptosis-inducing ligand	TRAIL
Unc-51-Like Kinase ½	ULK1/ULK2
UV irradiation resistance-associated tumor suppressor gene	UVRAG
Vascular endothelial growth factor receptor	VEGFR
